# Maternal Sedentary Behavior and Breast Milk Extracellular Vesicle MicroRNA Across Pregnancy: A Pilot Study

**DOI:** 10.3390/nu18193293

**Published:** 2026-10-08

**Authors:** Beatriz B. Elliott, Kara M. Whitaker, Jacob Gallagher, Brooke Burnett, Ian J. Shofner, Karl Kerns, Elizabeth M. McNeill

**Affiliations:** 1 Department of Food Science and Human Nutrition, Iowa State University, Ames, IA 50011-1078, USA; bpereira@iastate.edu (B.B.E.); brooke-burnett@uiowa.edu (B.B.); 2 Department of Health, Sport, and Human Physiology, University of Iowa, Iowa City, IA 52242-1111, USA; kara-whitaker@uiowa.edu; 3 Department of Kinesiology, Iowa State University, Ames, IA 50011-4008, USA; jgall13@iastate.edu; 4 Department of Animal Sciences, Iowa State University, Ames, IA 50011-3150, USA

**Keywords:** extracellular vesicles, microRNA, breast milk, sedentary behavior, pregnancy, lactation, infant development

## Abstract

Background/Objectives: Human breast milk (HM) is the gold-standard nutritional source for infants, delivering not only macro- and micronutrients but also bioactive components, including extracellular vesicles (EVs) enriched with microRNAs (miRNAs) that regulate immune, metabolic, and neurodevelopmental programming. Maternal lifestyle, including nutrition, is known to shape milk composition, yet it remains unknown whether maternal physical activity modulates the bioactive miRNA cargo of HM EVs. Methods: This pilot study examined associations between moderate-to-vigorous physical activity (MVPA; ≥3.0 metabolic equivalents) and prolonged sedentary behavior (SED60+; total sitting/lying minutes of bouts ≥60 min) with EV-enriched miRNA expression in HM collected in the early postnatal period (*n* = 11). Maternal activity was measured using a thigh-worn activPAL monitor across pregnancy trimesters and days 10–18 postnatal; HM was collected at day 18 postnatal. The EV-enriched fraction from skim milk was isolated and small RNA sequencing identified 691 unique miRNAs. Results: Principal component analysis showed that EV-enriched miRNA profiles were nominally most associated with prolonged sedentary behavior during the first two trimesters, though these associations did not survive correction for multiple testing. Differential expression analysis identified nine sedentary-associated miRNAs in trimester two, with smaller signals in trimesters one and three. Pathway enrichment revealed overrepresentation of genes involved in RNA regulation and post-transcriptional control, cell adhesion and cytoskeletal organization, and growth and nutrient signaling. Conclusions: Together, these findings suggest that maternal sedentary behavior during early and mid-pregnancy may be associated with candidate variation in HM EV-miRNA composition, offering a preliminary molecular link between prenatal behaviors and early-life programming that warrants replication in larger, independent cohorts.

## 1. Introduction

The World Health Organization (WHO) [1], the American Academy of Pediatrics (AAP) [2], and the U.S. Centers for Disease Control and Prevention (CDC) [3] recommend at least six months of exclusive breastfeeding to ensure infants receive the full health benefits of human breast milk (HM), supported by observational evidence. HM provides essential nutrients, including healthy fats, proteins, vitamins, and bioactive components, such as human milk oligosaccharides (HMOs). Infants who are fed breast milk have reduced neonatal mortality [4], gastrointestinal infections [5], and infectious diseases [6]. Moreover, there are additional benefits beyond disease prevention, including improvements in cognitive development [7,8,9]. Consequently, considerable attention has focused on HM components and mechanisms underlying these effects.

One component of growing interest is microRNAs (miRNAs) [10]. miRNAs are small, non-coding RNAs (18–25 nucleotides) that regulate biological processes through post-transcriptional repression of mRNA translation, primarily by binding the seed region to the 3′ untranslated region of target mRNAs. HM contains miRNAs involved in immunity, neurodevelopment, and metabolism [11]. Notably, milk EV miRNAs resist RNAse treatment and low pH [12,13,14], indicating stability in the acidic gut and bioavailability to infants. This resistance may result from HM’s high lipid content and transport within extracellular vesicles [15].

Extracellular vesicles (EVs) are lipid-bilayer-enclosed nanoparticles secreted by cells that play a critical role in intercellular communication. EVs are heterogeneous and broadly classified into exosomes, microvesicles, and apoptotic bodies based on size, biogenesis, and cellular origin [16]. Evidence indicates that HM EVs carry miRNAs and can interact with epithelial cells in the infant gut [14]. This highlights the need for mechanistic research on the role of HM miRNAs transported by EVs in infant health and development. HM EV miRNAs represent a compelling nutrigenomic signal: a maternal behavior-sensitive, biologically active component of the nutritional matrix infants consume daily.

An important research avenue is understanding behavioral influences on HM miRNA, such as maternal physical activity. Strong evidence supports the benefits of exercise for maternal health outcomes [17,18,19], yet its impact on breast milk composition remains largely unexplored. Existing studies suggest maternal physical activity may positively influence infant outcomes, including neurodevelopment [20], cortical structure [21], and birthweight [22]. For example, Domingues et al. (2014) reported slightly higher IQ at 48 months among children of active mothers, and maternal prenatal physical activity during pregnancy was positively associated with Battelle Developmental Inventory scores at 12 months [20]. Sedentary behavior may also affect infant health outcomes [23], though mechanisms remain unclear. HM miRNAs may represent one pathway through which maternal activity influences infant development.

To address the gap in understanding how maternal activity influences HM miRNA expression, this study examines the impact of maternal physical activity and sedentary behavior during pregnancy and the early postnatal period on miRNA profiles in the HM EV-enriched fraction. Maternal activity was measured across each trimester and early postnatal period using device-based monitoring, while HM samples were analyzed for EV-enriched miRNA composition.

We hypothesize that healthier 24 h movement behaviors, defined by greater active time and less sedentary time, will influence HM EV-enriched miRNA profiles targeting developmental pathways.

## 2. Methods

**Figure 1:**
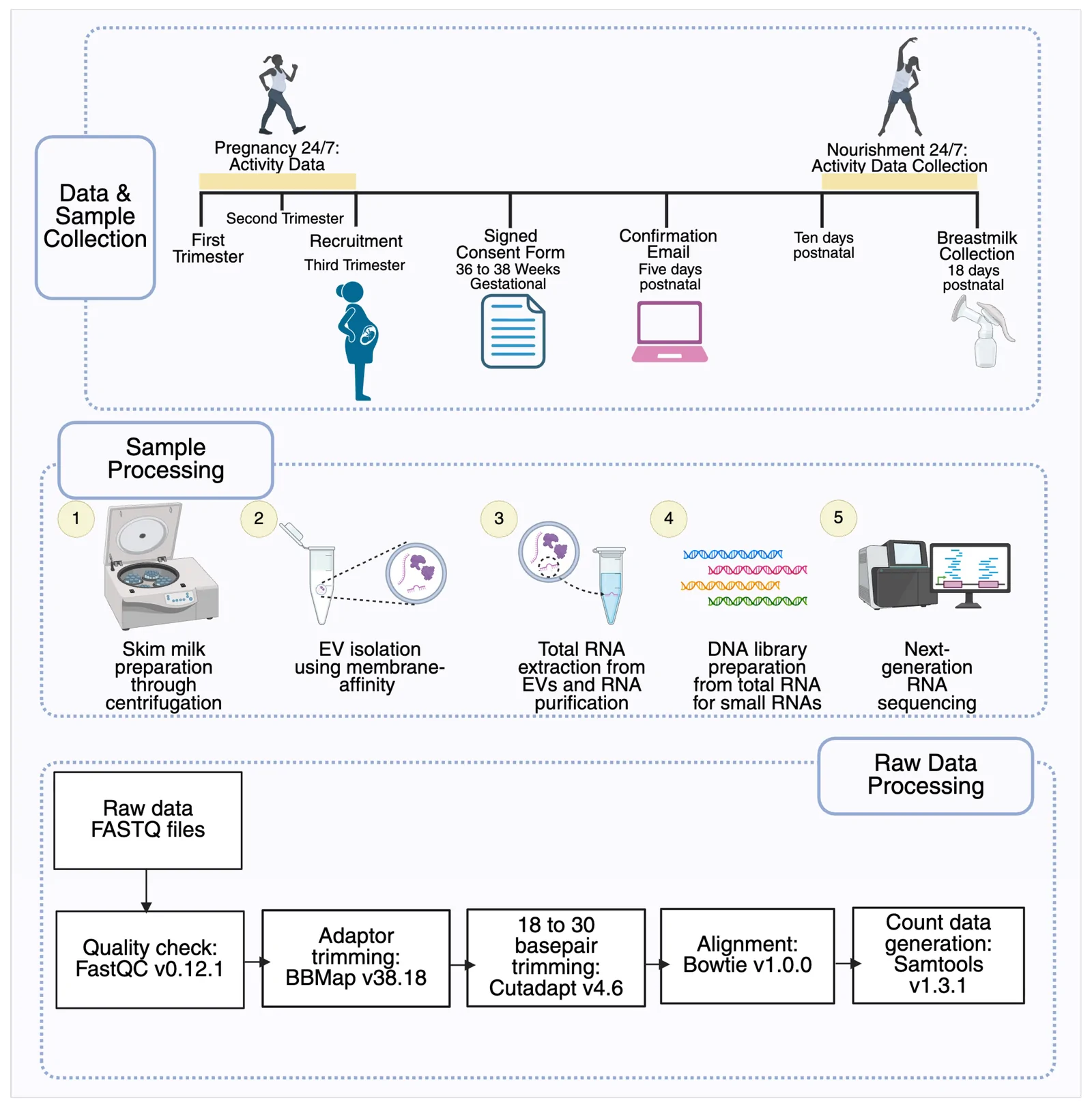
Study workflow: recruitment, breast milk collection, EV isolation, library preparation, and RNA sequencing. Participants were recruited in the third trimester, provided informed consent, and completed activity monitoring across pregnancy and postnatally. Breast milk was collected at 18 days postnatal. Samples underwent sequential processing: (1) skim milk preparation by centrifugation, (2) EV isolation using membrane-affinity methods, (3) total RNA extraction and purification, (4) small RNA library preparation, and (5) next-generation sequencing on an Illumina NovaSeq platform. Raw sequencing data were processed in a Linux environment: FASTQ files were merged after confirming no lane effects, quality-checked with FastQC, adapter-trimmed using BBMap, size-filtered (18–30 bp) with Cutadapt, and aligned to mature human miRNAs (miRBase) using Bowtie, and count matrices were generated with Samtools. Created in BioRender. Pereira, B. (2026) https://BioRender.com/15d52ur.

A graphical summary of the study workflow, including breast milk processing, EV isolation, RNA sequencing, and raw RNA sequencing data processing, is provided in Figure 1 to illustrate the methodological pipeline.

### 2.1. Participant Selection and Ethics

Participants were recruited from the multi-site Pregnancy 24/7 Cohort Study (NCT04749849), details of which have been previously reported [24]. The objectives of the larger study were to examine the associations between sedentary behavior, sleep, and the 24 h activity cycle and adverse pregnancy outcomes. Participants in the present study were a subset of women from the University of Iowa site.

All participants provided written informed consent between 36 and 38 weeks of gestation, prior to data collection beginning ten days postnatal. By signing the consent form, participants authorized collaborators at the University of Iowa to release de-identified prenatal data collected through Pregnancy 24/7 for analysis. A follow-up email was sent five days postnatal to confirm continued interest in participation. The study received ethical approval from both the Iowa State University Institutional Review Board and the University of Iowa Institutional Review Board prior to recruitment and data collection.

Eligibility criteria for the present study included being in the third trimester of pregnancy, aged 18–45, planning to breastfeed, owning a breast pump, having a singleton pregnancy, and giving birth at the University of Iowa Hospitals and Clinics. Additionally, women who experienced a multiple gestation birth, cesarean section, or adverse pregnancy outcome, including preeclampsia and preterm birth (defined as birth <37 weeks of gestation), were excluded to eliminate potential confounding factors. However, maternal-specific medical conditions, such as gestational diabetes or kidney stones, did not disqualify participants from the study. The University of Iowa collaborators confirmed participant eligibility via medical chart review once participants delivered.

### 2.2. Maternal Physical Activity Collection and Processing

Maternal activity data from Pregnancy 24/7 cohort participants recruited specifically for this ancillary study were analyzed. Details on prenatal activity data collection and processing are available elsewhere [24]. Briefly, activity data were collected during the first (10–12 weeks), second (20–22 weeks), and third (32–34 weeks) trimesters using an activPAL3 micro (Pal Technology, Glasgow, UK) monitor worn on the anterior right thigh for nine consecutive days. An Actiwatch device was worn on the wrist, and a sleep diary was kept to enhance accuracy and aid in sleep assessment.

Specific to the present study, postnatal data were collected over nine days (seven full, two partial) between days 10 and 18 postnatal. Participants wore the activPAL3 device secured with a waterproof dressing, and daily activity was recorded alongside a sleep log documenting wake time, rise time, bedtime, sleep attempt time, and any monitor removal exceeding ten minutes.

As previously described [25,26], activPAL3 data were exported using the VANE algorithm within the PAL analysis software (PALtechnologies, v8.11.1.63). Day segments, sleep periods, and non-wear times were identified through a custom R script informed by sleep diary entries. When discrepancies greater than 30 min occurred between diary-reported sleep times and activPAL3 data (e.g., activity detected during a sleep window), corrections were made using graphical representations of the activPAL3 output. Waking behaviors were summarized, and MET values derived from activPAL3 outputs, along with postural classifications, were used to quantify MVPA, LPA, and sedentary time.

We focused on two key measures: prolonged sedentary behavior, defined as total sitting/lying minutes of bouts lasting ≥60 min, and moderate-to-vigorous physical activity (MVPA), defined as activity ≥3.0 metabolic equivalents. For sedentary behavior, we considered the average number of minutes in sedentary bouts exceeding 60 min, consistent with WHO recommendations to interrupt prolonged sitting to reduce health risks [27]. During prenatal and postnatal periods, women often experience extended sedentary time due to pain, infant feeding, and caregiving, making bouts lasting ≥60 min a meaningful indicator of prolonged sedentary behavior. The MVPA definition aligns with established guidelines recommending 150 min per week of moderate-intensity activity [27].

### 2.3. Breast Milk Collection and Pre-Processing

On the morning of postnatal day 18, breast milk was collected via an electric pump following nipple cleansing with sterile ethanol. Participants first emptied the breast, allowed it to refill (about 2 h), and collected milk from the breast with the highest output. Initial volumes varied based on individual availability and were collected into a pre-sterilized 160 mL bag. Samples included all components of mature milk, including foremilk and hindmilk, and were frozen immediately at −18 °C and stored at −80 °C at the University of Iowa until transfer, with pick-up occurring within 12 h of collection. Samples were transported frozen. Upon transfer to Iowa State University, samples were thawed on ice, aliquoted, diluted in 1× PBS (Phosphate-Buffered Saline), centrifuged at 400× *g* for 10 min at 4 °C to remove cells, and stored at −80 °C until extraction, in 2.5 mL aliquots. Extracellular vesicle isolation and miRNA extraction were completed within five weeks of collection to preserve sample integrity. The total number of freeze–thaw cycles was limited to two [28].

### 2.4. Extracellular Vesicle Isolation and RNA Extraction

A quantity of 2.5 mL of each HM sample was thawed on ice. Once thawed, it was centrifuged at 720× *g* for 20 min to separate the milk fractions. Fat globules were eliminated by aspiration, and the skim milk fraction was collected by gently pipetting into a new RNAse-free tube. The skim milk fraction was then centrifuged at 720× *g* for an additional 20 min for further purification, and any remaining fat was again removed by aspiration.

For EV isolation, the Qiagen exoEasy Maxi kit (Qiagen, Cat. No. 76064; Hilden, Germany) was used according to the manufacturer’s instructions without any modifications, using 2 mL of the skim milk fraction. The EVs were eluted in buffer XE. RNA extractions were performed immediately after EV isolation. Total RNA was isolated from 200 µL of isolated EVs using the Qiagen miRNeasy Serum/Plasma kit (Qiagen, Cat. No. 217184; Hilden, Germany) following the manufacturer’s instructions without alteration. RNA was eluted with RNAse-free water. To facilitate downstream analysis, the total RNA eluate was further purified and concentrated using the RNA Clean & Concentrator™ kit (Zymo Research, Cat. No. R1015; Irvine, CA, USA) according to the manufacturer’s instructions, including DNase treatment. RNA concentrations and purity were verified using a Qubit with RNA HS reagents (Thermo Fisher Scientific, Cat. No. Q32880; Waltham, MA, USA) and the Nanodrop (Thermo Fisher Scientific, Wilmington, DE, USA) after the extractions.

### 2.5. Transmission Electron Microscopy

Extracellular vesicles isolated from human milk (1 µL) were fixed using a 50% stock solution of glutaraldehyde. A total of four samples were randomly selected from the sample pool for analysis. EVs were applied to carbon-coated copper grids, then negatively stained with 0.5% uranyl acetate. The grids were subsequently blot-dried and air-dried. Images were captured using a 200 kV JEOL 2100 scanning/transmission electron microscope (JEOL Ltd., Akishima, Tokyo, Japan), equipped with a Gatan CCD camera (Gatan Inc., Pleasanton, CA, USA) at the Roy J. Carver High-Resolution Microscopy Facility at Iowa State University.

### 2.6. EV Surface Cargo Labeling

EV surface cargo was measured using the Cellarcus vFC™ kit (Cellarcus Biosciences, San Diego, CA, USA) according to vFC Protocol 2. Briefly, isolated EVs were labeled with vFRed™ membrane stain and anti-human tetraspanin cocktail targeting CD9, CD63, and CD81, conjugated to PE (Cellarcus Biosciences), in Cellarcus Staining Buffer. Samples were incubated for 1 h at room temperature in the dark. The following controls were included: buffer only (background control), Lipo100 synthetic liposomes (negative control) buffer with all reagents (reagent background control), and Cellarcus EV Reference Preparation (positive control).

### 2.7. ImageStream Acquisition

Labeled samples were acquired on an ImageStream^®^X Mark II imaging flow cytometer (Cytek Biosciences, Fremont, CA, USA) using Cellarcus vFC Assay IDEAS Template for ImageStream, modified for a 5 min acquisition time. Instrument settings were configured as per Cellarcus Protocol A recommendations: all fluorescence excitation lasers at maximum power, SSC laser (Ch12) at 0.75 m, brightfield channels off, magnification set to 60×, high-gain mode enabled, and fluidics set to low speed/high sensitivity.

### 2.8. Vesicle Immunophenotyping and Quantification

Raw image files (.rif) were analyzed in IDEAS^®^ software v6.4 using the Cellarcus vFC Assay IDEAS Template for ImageStream. A hierarchical gating strategy (vFRed Area; No Speedbeads; Vesicles; vFRed+ Ab+) was applied, with the double-positive gate defined as a rectangular region in the upper-right quadrant of the Ch05 (vFRed) vs. Ch03 (antibody) scatter plot, positioned to exclude background signal. The percentage of vFRed+ vesicles that were antibody-positive (%Gated), particle concentration (Objects/mL), and mean fluorescence intensity (MFI) were extracted from IDEAS. Each biological sample was acquired in technical duplicate, and values were averaged prior to analysis.

### 2.9. Vesicle Diameter Calibration

Vesicle diameter was determined in FCS Express™ (v7.32.0070, De Novo Software) using the Cellarcus vFC™ Vesicle Size Calibration layout for ImageStream. Time, vFRed-H vs. -A, and Vesicles gates were re-established using the Lipo100™ synthetic vesicle standard, and a linear regression between vFRed-A fluorescence intensity and known particle surface area was used to calculate equivalent spherical diameter for each gated vesicle event. This calibration was applied uniformly to all breast milk samples and controls. Median diameter, geometric mean, and coefficient of variation were extracted for each sample and technical replicate, and replicate values were averaged.

### 2.10. Library Preparation, Size Selection, and Next-Generation Sequencing

Libraries were prepared using the NEBNext Multiplex small RNA library kit (New England Biolabs, Ipswich, MA, USA) for Illumina, with adjustments for low RNA concentrations, including diluting 1 µL the 3′ SR Adaptor and 5′ SR Adaptor, and 17 PCR cycles. A total of 300 ng RNA was used as input, and each sample was assigned an index for pooled sequencing. Total DNA library concentrations were quantified with the Qubit™ dsDNA HS Assay Kit (Thermo Fisher Scientific, Cat. Nos. Q32851 and Q32854), and library quality was verified using the Agilent 2100 Bioanalyzer HS DNA assay (Agilent Technologies, Santa Clara, CA, USA). Small RNA size selection was performed using the Monarch PCR & DNA Cleanup Kit (New England Biolabs, Ipswich, MA, USA; Catalog No. T1130) with 6% TBE gel and 1× TBE buffer with 0.5× EDTA at pH 8.0. A total of 15 µL of the prepared library was loaded into the wells, and the gel was run at 120 V for approximately 40 min. Bands within the 130–150 base-pair range were isolated from the gel and further purified using a gel filtration column. The DNA pellet was then resuspended in 12 µL of TBE buffer. The purified libraries were quantified using the Qubit™ dsDNA HS Assay (Thermo Fisher Scientific, Waltham, USA), and the final library quality was verified with the Agilent 2100 Bioanalyzer HS DNA assay (Agilent Technologies, Santa Clara, USA). Finally, the libraries were normalized and pooled into a single tube, then submitted for sequencing on the NovaSeq 6000 (Illumina, San Diego, USA) with a 100-cycle, SP, full-flow cell at the DNA Facility at Iowa State University.

### 2.11. RNA Sequencing Data Processing

RNA sequence data were received as a FASTQ file. The data were processed using a Unix-based operating system. Two flow cell lanes were used to generate reads from the same DNA library pool using multiplexed samples, and the appropriate (participant-matched) FASTQ files were combined after confirmation that there were no batch effects due to lane separation. Quality control for read distribution, base quality, and over-represented sequences was performed with FastQC v0.12.1. Adaptor removal was carried out using BBMap v38.18, with parameters set to allow two mismatches, trim the right end of reads (corresponding to 3 prime), and remove the small RNA 3 prime adaptor sequence “AGATCGGAAGAGCACACGTCTGAACTCCAGTCAC.” Cutadapt v4.6 was then used to eliminate sequences that were larger than 30 base pairs and smaller than 18 base pairs. Next, the trimmed reads were aligned to human mature miRNAs using the miRBase v22 mature miRNAs file (mature.fa) with Bowtie v1.0.0, allowing for zero mismatches in the seed region. Total counts were generated using Samtools v1.3.1.

### 2.12. Statistical and Bioinformatics Analysis

**Figure 2:**
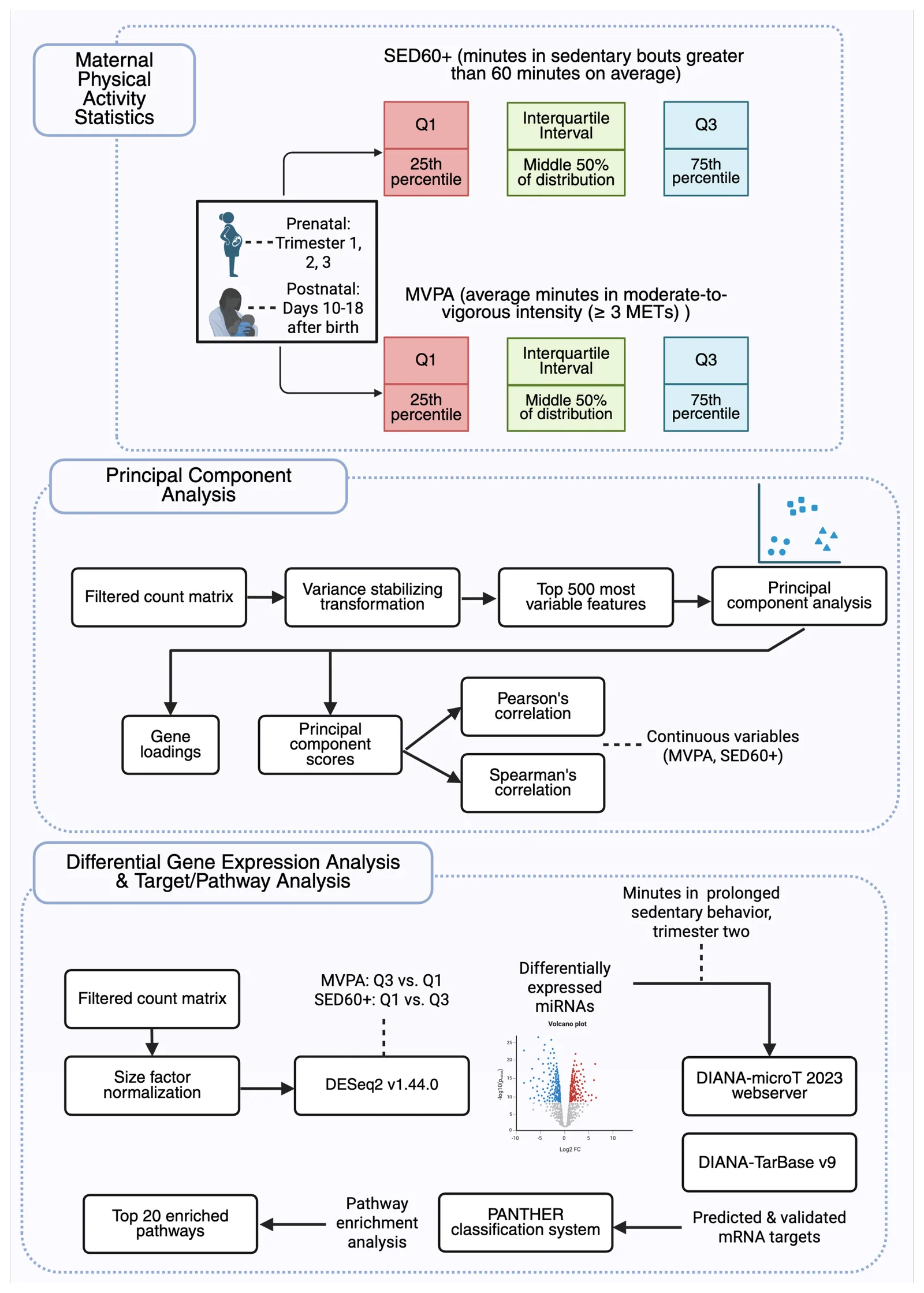
Overview of statistical method for physical activity characterization, EV-miRNA analytic workflow, and downstream target/pathway analysis. Maternal activity metrics were calculated separately for prolonged sedentary behavior (SED60+; minutes spent in sedentary bouts ≥60 min) and moderate-to-vigorous physical activity (MVPA; minutes spent at ≥3 METs). Participants were categorized into quartiles (Q1 in red, interquartile interval in green, and Q3 in blue) for each metric across the prenatal trimesters (T1–T3) and the postnatal period (days 10–18). Principal component analysis was performed, and PCA outputs (component scores and gene loadings) were correlated with continuous activity measures using Pearson’s and Spearman’s correlation coefficients. Differential expression analysis was conducted with DESeq2 v1.44.0, comparing Q3 vs. Q1 for MVPA and Q1 vs. Q3 for SED60+. Differentially expressed miRNAs were used to identify predicted and experimentally validated mRNA targets using DIANA microT and DIANA-TarBase, followed by Gene Ontology biological process enrichment using the PANTHER classification system. The top 20 enriched pathways were selected based on significant (FDR < 0.05) highest fold enrichment. Created in BioRender. Pereira, B. (2026) https://BioRender.com/7q0tljc.

Figure 2 summarizes the analytical workflow, including global miRNA expression analysis via VST and PCA, differential expression testing with DESeq2, and downstream target prediction and pathway enrichment using DIANA-microT (DIANA-microT 2023 web server), DIANA-TarBase v9, and PANTHER v18.0.

Maternal activity variables (prolonged sedentary minutes and MVPA) were categorized into lower (Q1, ≤25th percentile), interquartile interval (25th–75th percentile), and upper (Q3, ≥75th percentile) groups using sample-specific quartile cut points. Quartiles were derived in R using the quantile () function and were used to emphasize biologically meaningful contrasts between behavioral extremes, consistent with the exploratory design and limited sample size.

Maternal activity quartile cut points were applied to the 11 participants with available breast milk omics data. The activity quartiles were previously evaluated using both the original cohort (n = 13) and the sequencing analytic sample (n = 11); all results reported here reflect the final analytic sample (n = 11).

To explore global patterns in miRNA expression and identify sources of variation across samples, a variance-stabilizing transformation (VST) was applied to the filtered count matrix (minimum of five counts across at least three samples) to address mean–variance dependence. Principal Component Analysis (PCA) was conducted using the prcomp() function in R (v4.4.0) on the transposed VST matrix, with centering and scaling enabled. The percentage of variance explained by each principal component (PC) was derived from the eigenvalues. PC scores were exported for subsequent statistical analyses, including correlations with continuous activity variables (Pearson’s r and Spearman’s rank correlation coefficient). Benjamini–Hochberg (BH) false discovery rate correction was applied within each 16-test family. To evaluate whether any single miRNA disproportionately drove a component, each miRNA’s percent contribution to PC1/PC2 was calculated as its square loading. Gene loadings were analyzed to identify the features that contributed most to PC1 and PC2.

To identify miRNAs associated with maternal activity, differential expression analysis was conducted on a Linux-based system using the DESeq2 v1.44.0 [29] package in R v4.4.0.

Given the small sample size and the exploratory nature of this study, we focused differential expression analyses on the extreme quartiles (Q1 and Q3) of maternal activity rather than modeling activity as a continuous variable. By emphasizing biologically meaningful contrasts between extremes, this approach maximizes signal detection and reduces variability introduced by mid-range behaviors. Sedentary behavior (total sitting/lying minutes in bouts ≥60 min) was designated as the primary exposure based on PCA results showing that prolonged sedentary time accounted for greater interindividual variability in EV-miRNA profiles than MVPA (as reflected by stronger associations with PC2 in early pregnancy). MVPA (≥3.0 metabolic equivalents) was therefore analyzed as a secondary exposure, with results reported in the Supplementary Materials.

For each variable, a negative binomial generalized linear model was constructed (e.g., ~quartile placement for sedentary behavior, trimester one) to assess differential expression using the default Wald test. Raw count data were normalized using DESeq2’s default size factor estimation method. Before analysis, miRNAs were filtered to include only those with at least five counts in at least three samples. Differential expression was considered statistically significant at a false discovery rate (FDR, Benjamini–Hochberg) ≤ 0.05. The number of samples per group is shown in Supplementary Table S1.

As sensitivity analyses, we evaluated the directional consistency and dispersion-dependence of quartile-identified miRNAs (padj < 0.05) using three complementary approaches. First, a continuous-exposure model (raw sedentary/MVPA values, full analytic sample, n = 11) was used to assess whether the direction of effect for each quartile-identified miRNA was consistent with the extreme-group comparison; this model was evaluated for direction of effect only and not treated as an independent test. Second, DESeq2 dispersion and size-factor estimation were repeated using all n = 11 participants (Low/Mid/High as a three-level factor), retaining only the Low-vs-High contrast, to evaluate whether significance depended on dispersion being estimated from the extreme quartiles alone (n = 6–7). Third, hsa-miR-148a-3p, the most abundant sequence in this dataset (31.6% of total reads), was removed from the count matrix prior to normalization and each model re-fit, to assess whether findings were dependent on its presence.

Because DESeq2’s default outlier correction is not recommended at three replicates per group [29], maximum Cook’s distance was extracted for each miRNA per comparison and the distributions were inspected visually. The 95th percentile of each comparison’s distribution is reported as a numerical anchor for this inspection, applied consistently across all 8 comparisons; miRNAs visually separated from the bulk of their comparison’s distribution are noted whether or not they crossed this specific value.

Significantly differentially expressed miRNAs identified through DESeq2 were further analyzed to explore their potential biological impact. Predicted miRNA–mRNA interactions were identified for each miRNA using the DIANA microT-CDS (2023) web server [30], applying a conservative interaction score threshold (≥0.80) to prioritize high-confidence, canonical target predictions. To further increase confidence in the inferred targets, predicted genes were cross-referenced with experimentally supported miRNA–mRNA interactions curated in DIANA-TarBase v9 [31]. The resulting non-redundant set of target genes was subjected to pathway overrepresentation analysis using the PANTHER Classification System (version 19.0) [32]. The background gene set consisted of all target genes predicted by DIANA microT-CDS across all miRNAs represented in the cohort. Pathway enrichment was assessed using Fisher’s exact test with Benjamini–Hochberg false discovery rate (FDR) correction, and pathways with adjusted *p*-values ≤ 0.05 were considered statistically significant. To emphasize the most robust biological signals, enriched pathways were ranked by fold enrichment, and the top 20 pathways were selected for visualization and reporting.

All code scripts, including RNA raw data processing, statistical analysis, and generated graphs, are available on the GitHub page [33].

## 3. Results

### 3.1. Participant Characteristics

**Figure 3:**
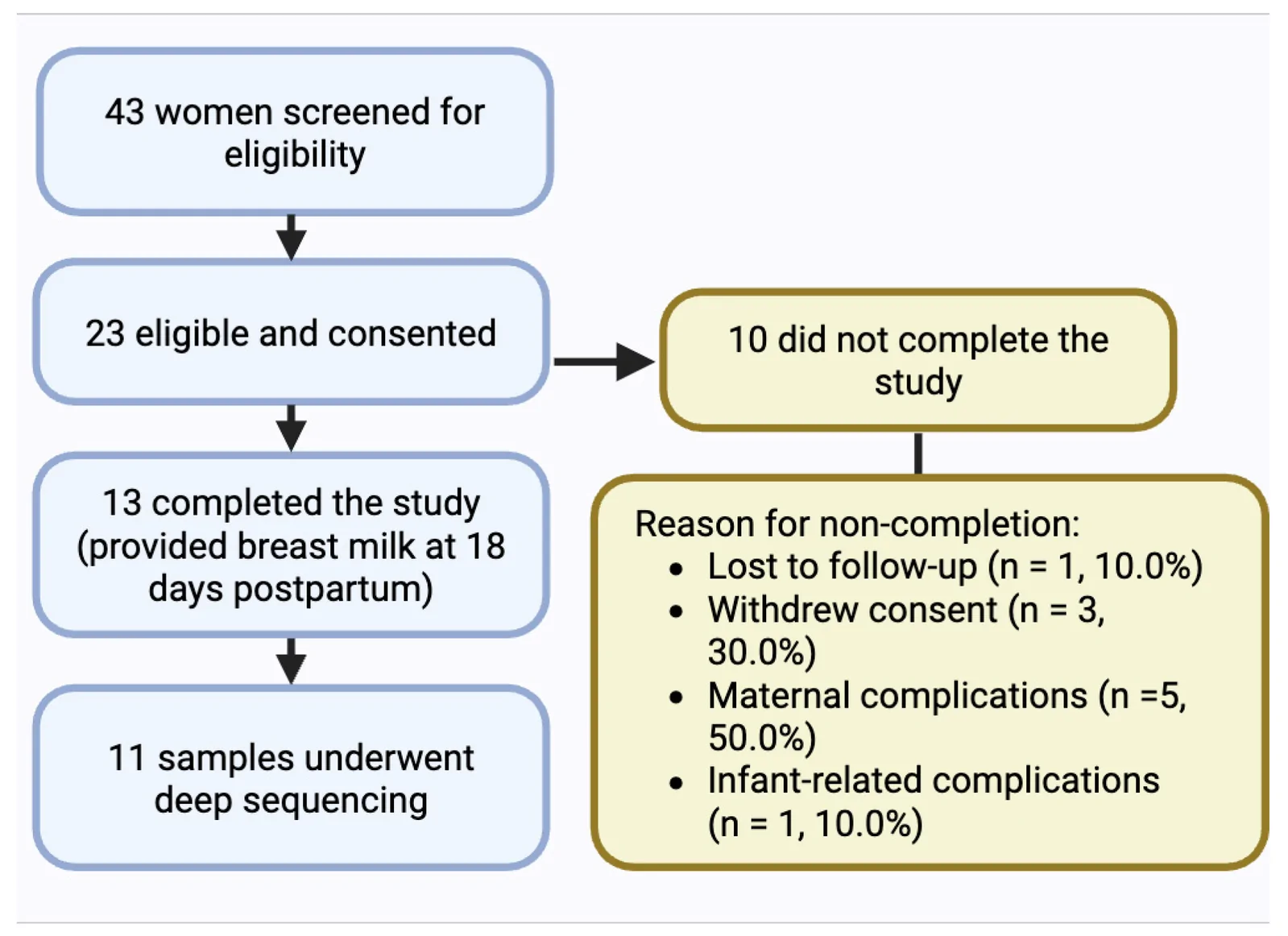
Study enrollment and completion flow chart. A total of 43 women were screened; 23 were eligible and consented. Of these, 13 completed the study and provided breast milk at 18 days postnatal. In total, 11 samples were subjected to deep sequencing. Created in BioRender. Pereira, B. (2026) https://BioRender.com/w9qgfhu.

Of the 23 women enrolled during the third trimester, 13 provided breast milk samples at 18 days postnatal. Reasons for non-completion included lost to follow-up (n = 1), withdrawal of consent (n = 3), maternal postnatal complications (n = 5; mastitis, n = 1; cesarean delivery, n = 4), and infant-related complications (IUGR; n = 1) (Figure 3). Demographic and clinical characteristics of the 13 participants who completed the study are summarized in Table 1. Due to study time constraints, 11 out of the 13 samples underwent deep sequencing.

### 3.2. Patterns in Prolonged Sedentary Behavior and MVPA

Prolonged sedentary time (minutes per day in ≥60-min bouts) showed variability across pregnancy, with lower values in the second trimester and a partial return toward first-trimester levels by the third trimester, most evident at the lower quartile, followed by a pronounced increase postnatal across all quartiles (Supplementary Table S2, Supplementary Figure S1). In contrast, MVPA generally declined over time, with decreases most evident in the median and upper-quartile values from early pregnancy through postnatal, while lower-quartile MVPA did not decrease during pregnancy but declined after delivery. Overall, these patterns indicate a shift toward greater sedentary behavior and lower physical activity levels, with the most substantial changes occurring in the postnatal period.

### 3.3. Proof-of-Concept Extracellular Vesicle Characterization

#### 3.3.1. Transmission Electron Microscopy

**Figure 4:**
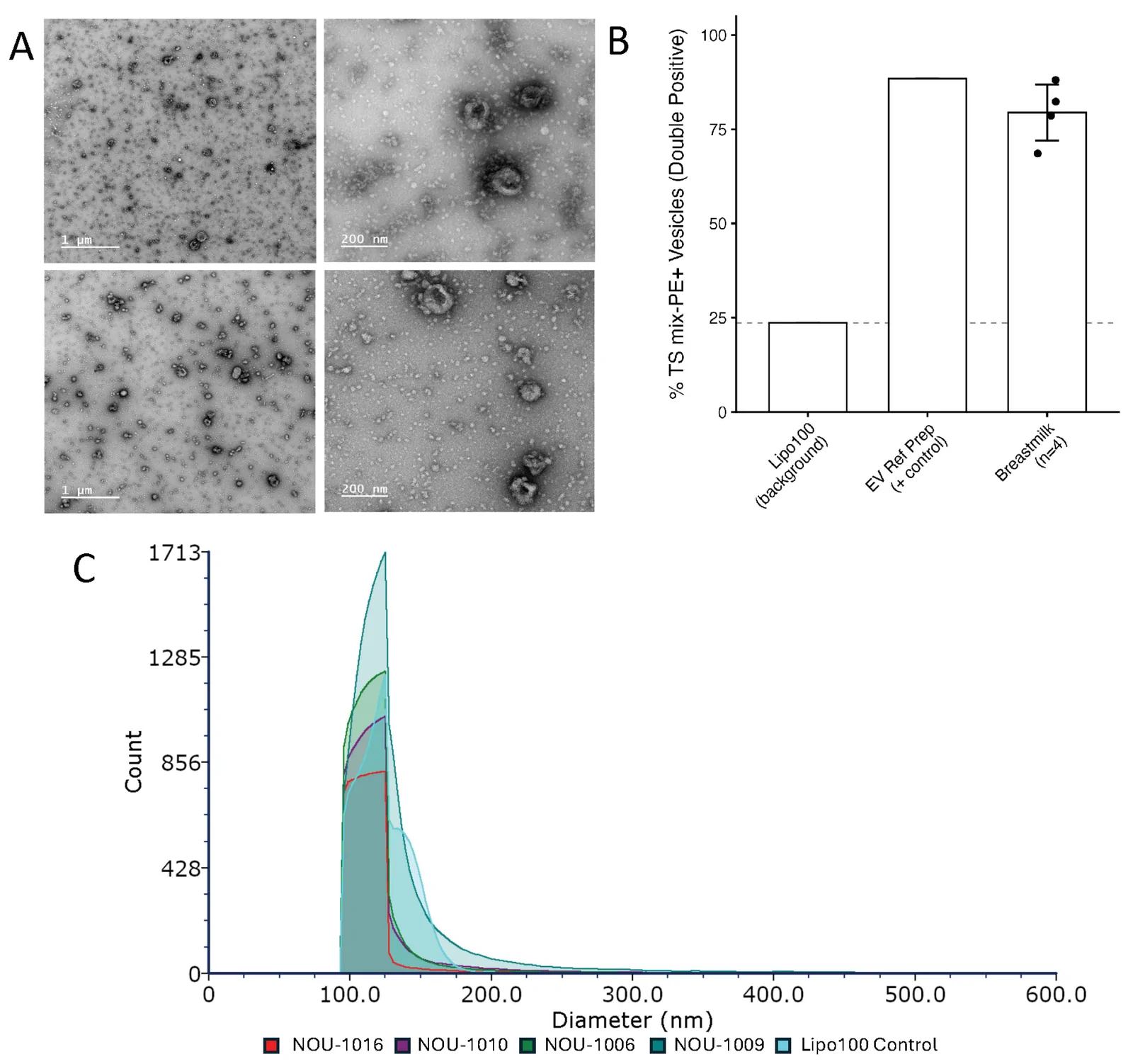
Characterization of breast milk-derived extracellular vesicle-enriched fraction. (**A**) Representative transmission electron microscopy (TEM) images of breast milk-derived EV-enriched fraction at low (scale bar = 1 µm) and high (scale bar = 200 nm) magnification. (**B**) Percentage of vFRed+ vesicles positive for tetraspanin mix-PE (CD9/CD63/CD81), shown for the Lipo100™ antigen-negative background control, the EV reference preparation (positive control), and breast milk EV-enriched samples (n = 4; individual points represent biological samples, bar denotes mean ± SD). Dashed line indicates the Lipo100™ background threshold. (**C**) Representative overlaid diameter histograms for the four breast milk EV-enriched samples and the Lipo100™ synthetic vesicle standard, generated in FCS Express™ following vFRed fluorescence calibration against the Lipo100™ standard.

TEM analysis confirmed the presence of intact, membrane-bound vesicles consistent with EV morphology (Figure 4A).

#### 3.3.2. EV Surface Cargo Detection and Vesicle Size by Flow Cytometry

Vesicle size was measured by vFC using vFRed membrane fluorescence calibrated against the Lipo100™ synthetic vesicle standard. All samples displayed narrow, right-skewed size distributions with median diameters of 96.0–103.3 nm (geometric mean: 100.4–115.2 nm; Figure 4C, Supplementary Table S3).

Tetraspanin surface marker expression (CD9/CD63/CD81-PE) was elevated across all breast milk samples relative to the Lipo100 antigen-negative background control (23.65% double-positive), with a mean of 79.4% ± 7.4% double-positive events across the four biological samples (range: 69.5–87.1%), comparable to the EV reference preparation (88.4%; Figure 4B, Supplementary Table S4). Together, these results confirm the isolation of appropriately sized, tetraspanin-positive extracellular vesicles from breast milk, consistent with expected EV characteristics.

Nonetheless, as membrane-affinity isolation methods do not fully exclude co-isolated impurities such as lipoproteins, particles are conservatively referred to as EV-enriched preparations throughout this manuscript, in accordance with MISEV guidelines.

### 3.4. EV-Enriched miRNA Expression Profiles and Sequencing Quality

**Figure 5:**
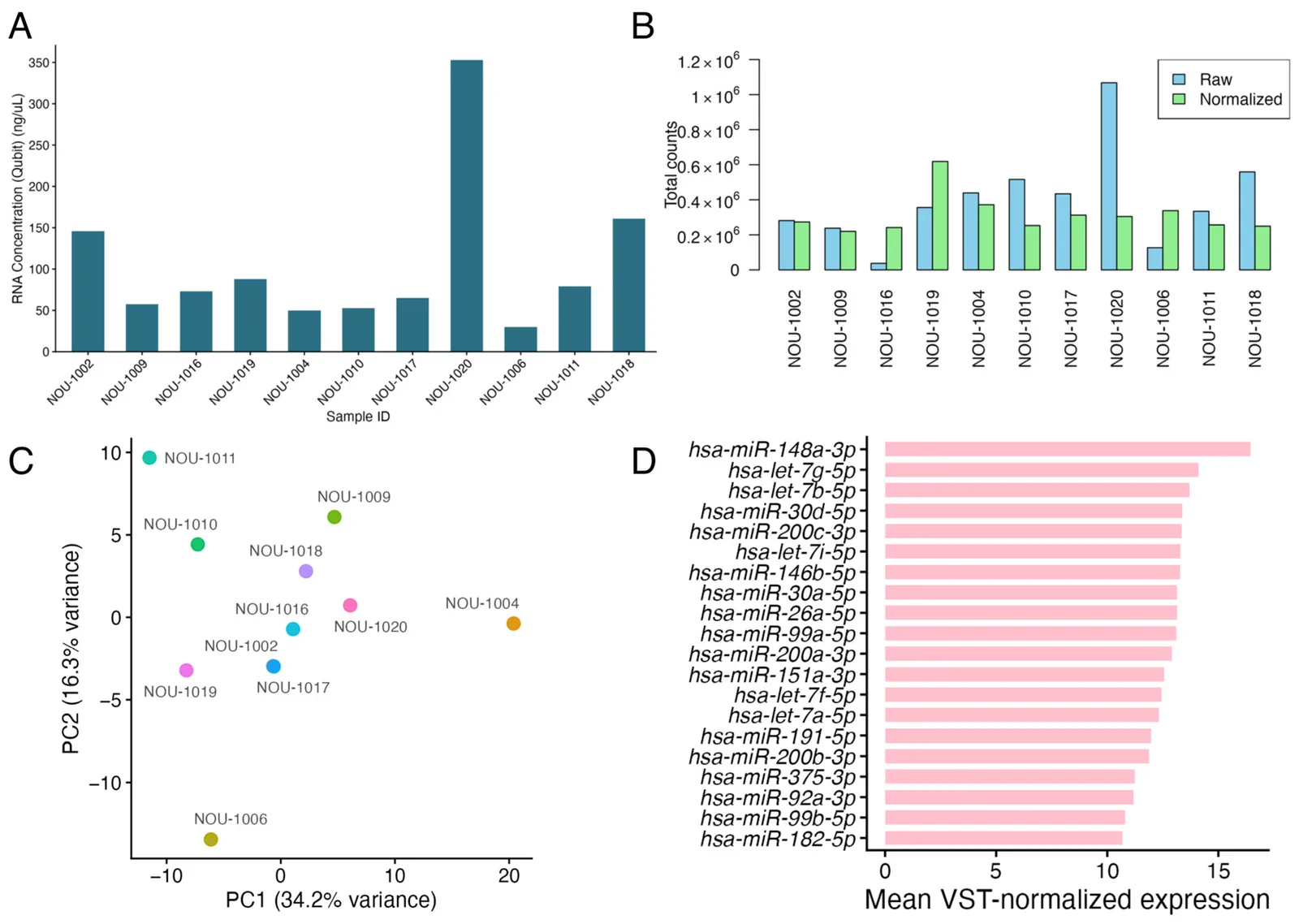
EV-enriched miRNA profiling across breast milk samples. (**A**) Total HM EV-enriched RNA concentration (ng/µL) measured for each sample, showing variability across donors. (**B**) Comparison of raw and normalized merged miRNA counts per sample, illustrating the effect of variance-stabilizing normalization used for subsequent analysis. (**C**) Principal component analysis (PCA) of normalized miRNA counts, with samples colored by ID; PC1 and PC2 explain 26.6% and 14.8% of the variance, respectively. (**D**) Bar plot of the top 20 most highly expressed miRNAs across all samples after normalization, ranked by mean expression (VST-normalized).

Total RNA yield varied across samples, with a median concentration of 73 ng/μL (range: 30–353 ng/μL) (Figure 5A), normalized to 300 ng for library preparation. Prior to merging sequencing lanes, correlations between lane one and lane two were assessed at multiple stages: raw read counts, post-trimming counts (adapter and size filtering), and final aligned counts (Figure S2A–C). Median raw reads were 38.1 million (lane 1) and 38.5 million (lane 2), with a perfect correlation (r = 1.00). After trimming, median reads were 3.61 million and 3.67 million, respectively, again with a correlation of 1.00. Final aligned counts showed a median of 219,140 (lane 1) and 179,963 (lane 2), with a high correlation (r = 0.995). Trimmed FASTQ files were merged, yielding a median of 7.2 million reads per sample. Aligned mature human miRNA counts had a median of 355,991 (Figure 5B).

Count data were normalized using the DESeq2 size factor estimation method to account for biological variability. A total of 691 unique miRNAs were detected; after low-count filtering (≥5 counts in ≥3 samples), 281 miRNAs were retained for PCA. Principal component analysis (PCA) revealed that PC1 accounted for 26.6% of the variance (Figure 5C), with samples NOU-1004 (−19.5), NOU-1011 (12.1), and NOU-1019 (10.9) contributing most strongly to this component. There was no obvious clustering based on sample identity. The most highly expressed miRNA was hsa-miR-148a-3p, comprising 31.6% of total reads, followed by hsa-let-7g-5p (7.87%) and hsa-let-7b-5p (4.96%). The top 20 most abundant miRNAs are shown in Figure 5D.

### 3.5. Exploratory Principal Component Analysis Indicates That Maternal Sedentary Behavior Is an Independent Source of Variability in PC2, Specifically in Early Pregnancy

**Figure 6:**
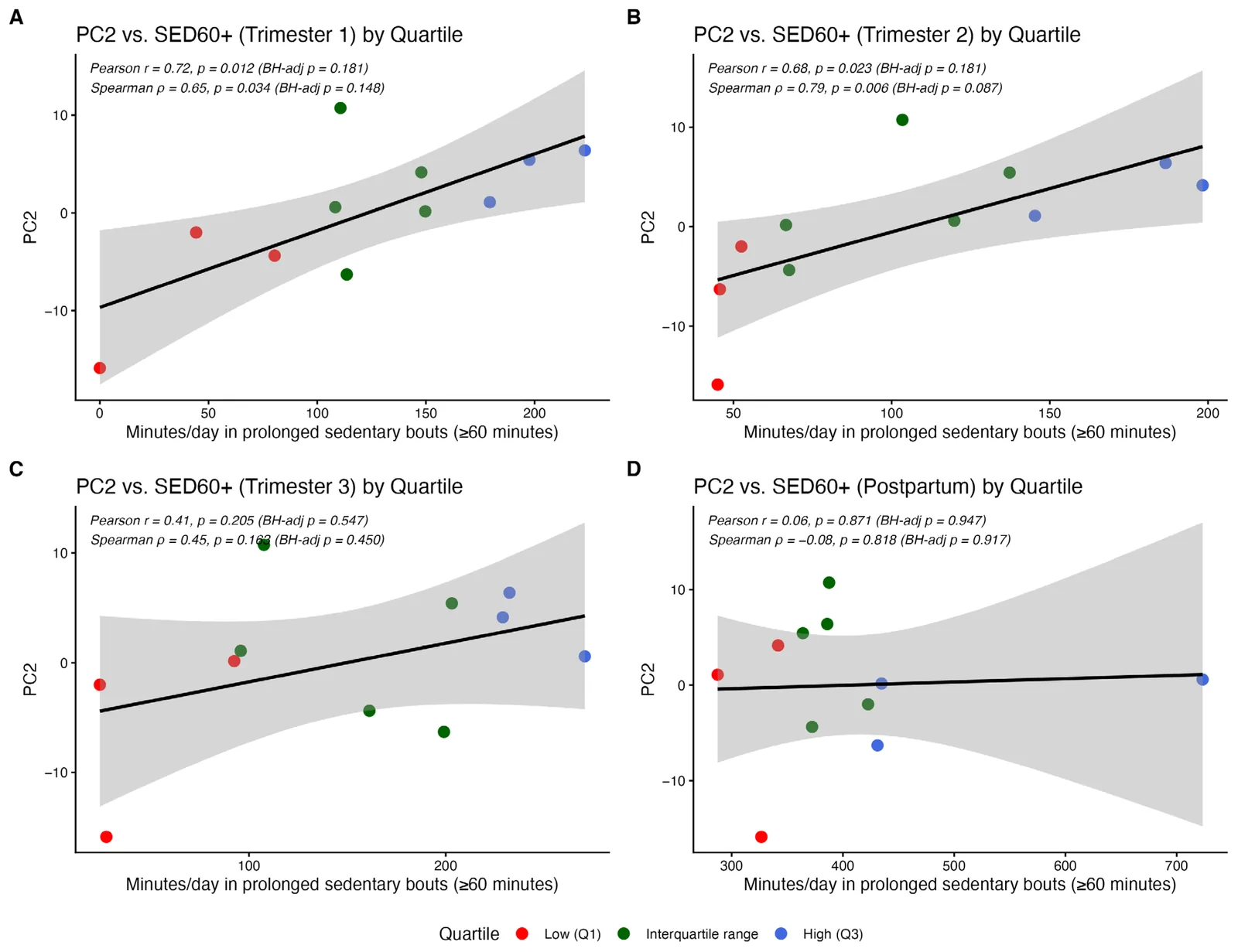
Associations between PC2 of EV-enriched miRNA expression and maternal physical activity patterns across pregnancy. Scatter plots with linear regression lines (black) and 95% confidence intervals (gray shading) showing the association between PC2 scores and average minutes/day in sedentary bouts ≥60 min for (**A**) trimester 1, (**B**) trimester 2, (**C**) trimester 3, and (**D**) postnatal. Points are colored by quartile group: Q1 (red), interquartile range (green), and Q3 (blue). Pearson’s correlation coefficient (r) and Spearman’s rank correlation coefficient (ρ) with corresponding *p*-values and BH-adjusted *p*-values are displayed in the upper-left of each panel. All analyses conducted in n = 11 participants.

PC2 of EV-enriched miRNA expression showed a consistent pattern of association with maternal sedentary behavior and MVPA, strongest in trimester 1 (sedentary: r = 0.72, ρ = 0.65; MVPA: r = −0.64, ρ = −0.65) and trimester 2 (sedentary: r = 0.68, ρ = 0.79; MVPA: r = −0.53, ρ = −0.73), attenuated by trimester 3 (sedentary: r = 0.41, ρ = 0.45; MVPA: r = −0.35, ρ = −0.35), and absent at postnatal (sedentary: r = 0.06, ρ = −0.08; MVPA: r = 0.09, ρ = 0.06) (Figure 6A–D; Table S5). No test reached padj < 0.05 after BH correction, with the trimester 2 sedentary behavior association trending towards significance (Spearman padj = 0.087). PC1 showed no such pattern.

hsa-miR-148a-3p, the most abundant sequence in this dataset, did not disproportionately drive PC2 (1.2% of loading) (Figure S3) or influence PCA correlational test results (Table S6).

Together, these findings suggest a consistent, hypothesis-generating pattern in which maternal activity in early pregnancy is associated with coordinated shifts in EV-enriched miRNA expression captured by PC2, diminishing as pregnancy progresses, that warrants further exploration.

### 3.6. Differential Expression Analysis Showed Trimester-Specific Patterns Linking Maternal Sedentary Behavior to EV-Enriched miRNA Profiles

**Figure 7:**
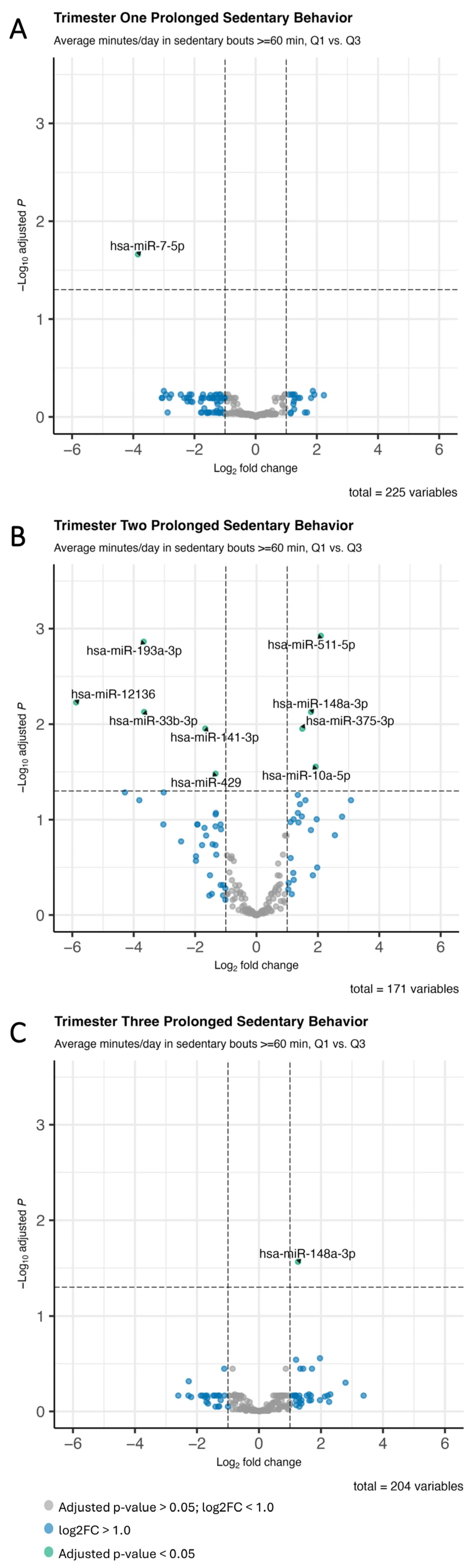
Differential expression of EV-enriched miRNAs by trimester-specific prolonged sedentary behavior. Volcano plots display log_2_ fold-change (x-axis) and −log_10_ *p*-values (y-axis) for EV-enriched miRNAs associated with prolonged sedentary behavior (minutes/day in sedentary bouts ≥60 min) comparing the lowest (Q1) and highest (Q3) quartiles within each trimester. (**A**) Trimester one (Q1 vs. Q3). (**B**) Trimester two (Q1 vs. Q3). (**C**) Trimester three (Q1 vs. Q3). Vertical dashed lines indicate ±1 log_2_ fold change (threshold = 1.0), and the horizontal dashed line marks the *p*-value threshold of 0.05. Points are colored by significance: grey = does not meet FDR or log_2_FC threshold; blue = log_2_FC only; teal = FDR and log_2_FC. Select miRNAs exceeding significance thresholds are labeled in each panel.

Prolonged sedentary behavior displayed the strongest associations in trimester two, where a total of nine miRNAs were differentially expressed, including both up- and down-regulated transcripts, with Q3 of SED60+ as the reference group (Table 2, Figure 7A–C). Trimester one had one downregulated miRNA, hsa-miR-7-5p, and only one sedentary-associated miRNA remained in trimester three, hsa-miR-148a-3p (upregulated). There were no differentially expressed miRNAs at postnatal for sedentary behavior. MVPA produced smaller but distinct associations overall, with one to three differentially expressed miRNAs detected per time point (Supplementary Table S7).

As sensitivity analyses, we evaluated whether quartile-identified associations were directionally consistent under a continuous-exposure model, whether significance was retained when dispersion and size-factor estimation used the full analytic sample (n = 11) rather than the extreme quartiles alone, and whether findings depended on the presence of the single most abundant miRNA, hsa-miR-148a-3p, in the count matrix (Supplementary Table S8); quartile-identified miRNAs were directionally consistent across all three checks and largely independent of hsa-miR-148a-3p, but showed reduced significance when dispersion was estimated from the full sample, indicating that these findings, while directionally robust, remain limited by sample size, or, alternatively, that effects of maternal activity behavior require extreme differences between SED60+ and MVPA, warranting follow-up in larger studies.

Normalized counts for differentially expressed miRNAs are provided in Supplementary Table S9 (all timepoints) and Figure S4 (trimester 2).

Of the 17 miRNAs reported as differentially expressed, two showed maximum Cook’s distances at or above the 95th percentile of their comparison’s distribution: hsa-miR-10a-5p in trimester 2 prolonged sedentary behavior and hsa-miR-7-5p in trimester 1 sedentary behavior (Table S10; Figure S5). These are interpreted with caution given the possibility of disproportionate influence from a single participant. Although hsa-miR-223-3p (MVPA postnatal) did not meet this threshold (maxCooks = 8.20 versus a comparison-specific 95th percentile of 9.18), visual inspection of its distribution showed clear separation from the remaining miRNAs in that comparison. This finding is therefore also interpreted with caution. The remaining 14 miRNAs showed Cook’s distances within the typical range for their comparison.

### 3.7. Pathway Enrichment for Trimester Two Sedentary Behavior

**Figure 8:**
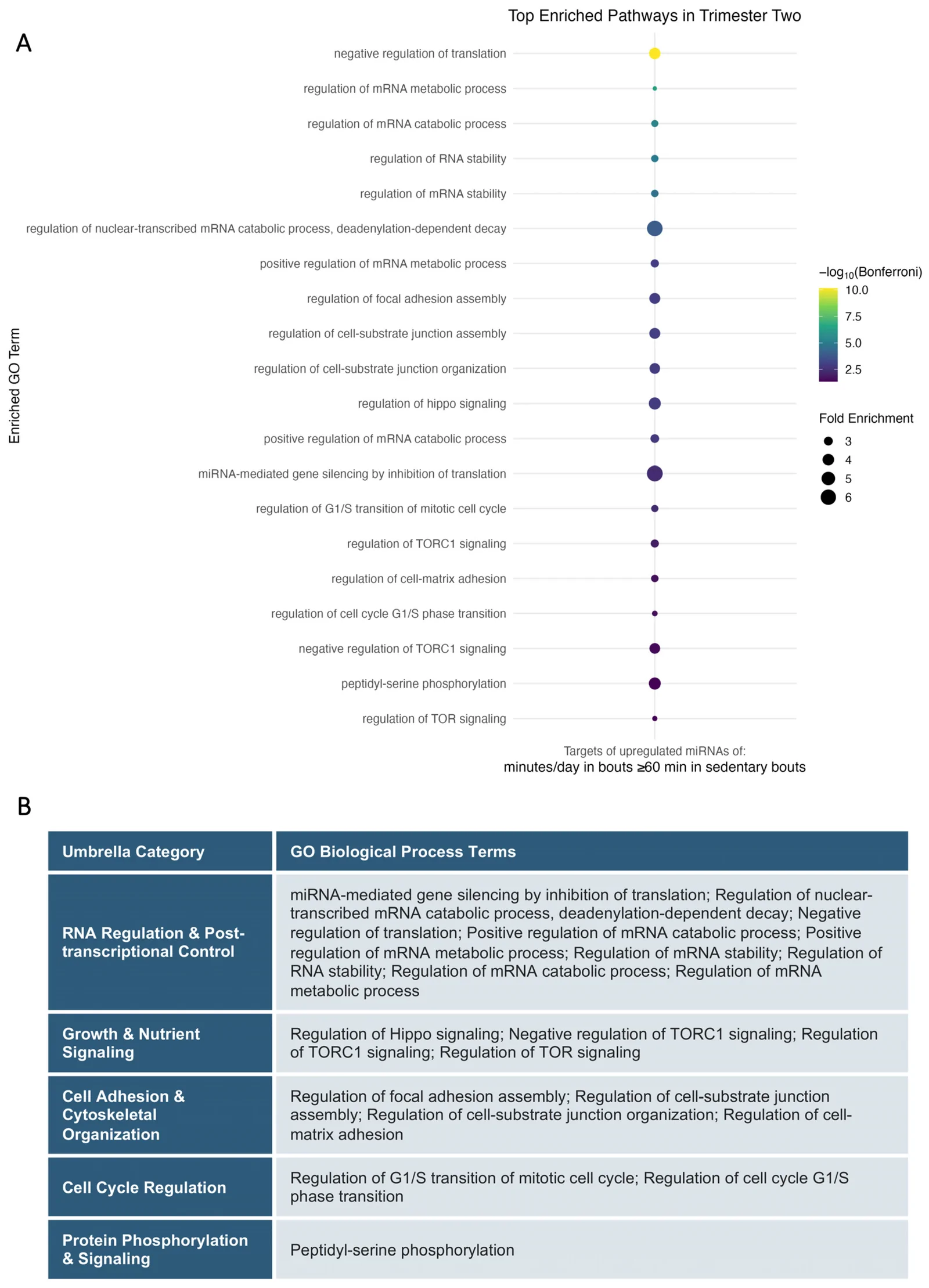
Pathway enrichment analysis of predicted targets of upregulated EV-enriched miRNAs associated with second-trimester sedentary behavior. Predicted mRNA targets of EV-enriched miRNAs positively associated with minutes/day spent in sedentary bouts ≥60 min during the second trimester were analyzed using PANTHER (GO Biological Process Complete). (**A**) Shown are the top 20 enriched GO terms based on fold enrichment, organized by significance (highest significance at the top). Each point represents a GO term, with point size corresponding to fold enrichment and point color indicating statistical significance (−log10 Bonferroni-corrected *p*-value). All displayed pathways passed multiple-testing correction. (**B**) Top enriched terms primarily involved RNA regulation and post-transcriptional control, cell adhesion and cytoskeletal organization, growth and nutrient signaling, cell cycle regulation, and protein phosphorylation.

Using PANTHER (GO Biological Process Complete), we identified a broad set of significantly enriched biological processes among predicted miRNA targets (FDRcorrected *p* < 0.05) in trimester two sedentary behavior, with the exclusion of hsa-miR-10a-5p, based on previously discussed caution on its interpretation. From these, the top 20 pathways were selected based on the highest fold enrichment for presentation (Table S11; Figure 8A). These top pathways were dominated by processes involved in RNA regulation and post-transcriptional control; cell adhesion and cytoskeletal organization; cell cycle regulation; and growth, nutrient, and phosphorylation-mediated signaling (Figure 8B).

Nine RNA-related pathways demonstrated strong enrichment, where the highest fold enrichment was seen for miRNA-mediated gene silencing by inhibition of translation (fold enrichment = 6.39, *p* = 4.33 × 10^−3^), and the most significantly enriched pathway was negative regulation of translation (3.86, *p* = 7.17 × 10^−11^).

A second major functional cluster involved growth and nutrient signaling, with four enriched pathways. Significantly enriched pathways included regulation of Hippo signaling (4.16, *p* = 1.47 × 10^−3^), negative regulation of TORC1 signaling (3.61, *p* = 2.97 × 10^−2^), regulation of TORC1 signaling (2.82, *p* = 1.17 × 10^−2^), and regulation of TOR signaling (2.38, *p* = 4.38 × 10^−2^). Additional enriched pathways were related to cell adhesion and cytoskeletal organization, cell cycle regulation, and protein phosphorylation.

## 4. Discussion

Our findings suggest that maternal activity patterns in early pregnancy, particularly prolonged sedentary behavior, are associated with candidate, hypothesis-generating variation in human milk extracellular vesicle-enriched miRNA profiles that warrants replication in larger, independent cohorts. Across the four timepoints measured, the most consistent associations emerged in the first and second trimesters, highlighting early and mid-pregnancy as windows of heightened sensitivity for HM EV-enriched miRNA variation. These results establish maternal activity during pregnancy as a potential modulator of HM miRNA composition, providing new insight into behavioral influences on breast milk bioactivity.

### 4.1. Maternal Activity in Early Pregnancy Is Associated with EV-Enriched miRNA Composition

Principal component analysis suggests a pattern in which maternal prolonged sedentary behavior in trimesters one and two showed the strongest nominal (uncorrected) association with variation along PC2, whereas MVPA showed weaker and more limited associations. Although preliminary, these early pregnancy effects are biologically plausible because mammary gland development accelerates during the first trimester [34], with extensive epithelial proliferation, vascular remodeling, and hormonal priming in preparation for lactation. Behavioral factors during this developmental window may influence systemic metabolic and inflammatory signals, thereby altering miRNA biogenesis, post-transcriptional processing, or EV packaging within the mammary gland.

Our observation that sedentary behavior in the second trimester showed the strongest nominal association with EV-enriched miRNA variation is conceptually consistent with prior work by Jones and colleagues (2022), who reported that longer periods of sedentary time during pregnancy were associated with faster increases in children’s BMI z-scores up to 2 years of age [35], with greater sedentary time in the first trimester specifically associated with an accelerated growth rate. We note, however, that this is a conceptual touchpoint rather than a direct correspondence. Jones et al. examined a different outcome in an independent cohort, and the two findings should not be read as validating one another. Both nonetheless point to early pregnancy as a window worth further study.

Notably, no association between sedentary behavior and EV-enriched miRNA composition was observed at the postnatal timepoint, despite postnatal sedentary time showing the largest absolute increase of any period measured. This may reflect a biological window that has already closed, where the developmental processes proposed above, mammary epithelial proliferation and hormonal priming in early pregnancy, are most active during gestation, so a behavioral exposure occurring after these processes have largely concluded may have a more limited capacity to shape EV-enriched miRNA composition. It may also be due to the general, consistently large, amount of time spent in prolonged sedentary behavior in the early postnatal period across samples.

Together, these findings indicate that early-pregnancy sedentary behavior may influence HM EV-enriched miRNA signatures in ways relevant to infant growth.

### 4.2. Comparison to Previous Research

Human milk contained 691 unique EV-enriched miRNAs, consistent with previous reports of high EV-enriched miRNA diversity and dominance of miR-148a-3p [36] and let-7 [36] family members. miR-148a-3p has well-established roles in immune regulation [37], cell differentiation [38], and metabolism [37,39], aligning with the known functions of HM miRNAs in supporting infant immunity and growth. Likewise, let-7 family members are known for their roles in developmental timing, immune modulation, and tumor suppression [40].

Because hsa-miR-148a-3p was both the single most abundant sequence in this dataset and a differentially expressed miRNA in more than one timepoint, we treat this recurrence with caution rather than as independent biological confirmation. High-abundance, evolutionarily conserved miRNAs such as miR-148a-3p and let-7 family members are consistently reported as dominant across human and mammalian milk EV-miRNA studies [36], so their frequent detection may partly reflect greater statistical power for detection at a high read depth or compositional properties inherent to relative-abundance sequencing data. To address this directly, we re-examined our findings after excluding hsa-miR-148a-3p from the count matrix in both the differential expression and PCA analyses; the results were materially unchanged. On this basis, we retained hsa-miR-148a-3p in the primary analysis given its limited influence on the overall findings and its established biological relevance to human milk and infant health.

Among trimester two sedentary-behavior miRNAs, several converge on metabolic and immune regulation. hsa-miR-375-3p (upregulated in less prolonged sedentary behavior) and hsa-miR-33b-3p (downregulated) have established roles in glucose and lipid metabolism, respectively [41,42], and hsa-miR-193a-3p (down) is notable for its paralog miR-193b-3p, which is elevated in prediabetes and normalizes with exercise intervention in humans [43]. hsa-miR-511-5p (up) is linked to macrophage polarization and inflammatory tone [44], though the functionally validated arm in the macrophage-polarization literature is miR-511-3p rather than the -5p arm detected here. Finally, hsa-miR-148a-3p (up) has documented gut-protective functions relevant to infant intestinal health [45].

A second theme touches on neuronal and epithelial development. hsa-miR-12136 (down) has been implicated in nervous system development in prior work [46], and hsa-miR-141-3p and hsa-miR-429 (both down) belong to a family governing epithelial differentiation [47].

Of note, predicted or literature-reported gene targets do not by themselves establish that these miRNAs act on infant neurodevelopment. Evidence for dietary milk miRNA bioavailability remains context-, species-, dose-, and method-dependent, and intestinal uptake of milk EV miRNAs is distinct from systemic bioavailability and functional gene regulation in the recipient infant [12,14,48]. These associations offer promising, hypothesis-generating leads for future mechanistic work. The convergence of trimester two candidates on metabolic–immune and neuro-epithelial developmental pathways is a coherent and biologically plausible pattern and provides a strong starting point for targeted validation in larger cohorts.

### 4.3. Biological Processes Enriched Among Sedentary-Associated miRNAs

Target prediction and PANTHER pathway enrichment indicated that sedentary-associated trimester two miRNAs regulate genes involved in five major biological domains: (1) RNA processing and post-transcriptional control; (2) cell adhesion and cytoskeletal organization; (3) growth and nutrient signaling, particularly the TOR/TORC1 and Hippo pathways; (4) cell cycle regulation; and (5) protein phosphorylation. The RNA-regulatory signal is notable given that miRNA biogenesis and EV packaging are themselves sensitive to cellular stress and metabolic status [49,50,51,52], while the growth/nutrient-signaling enrichment is plausible given that TOR/TORC1 directly senses cellular energy status. Together, these domains suggest candidate links to mammary and cellular growth regulation, but given the small sample size and the absence of functional validation, they should be treated as hypothesis-generating.

### 4.4. Importance and Implications

Our findings suggest that prolonged sedentary time, already recognized in pregnancy guidelines for its cardiovascular implications [19], may also be linked to subtle molecular changes during gestation, with potential implications for maternal and infant health. The trimester-specific EV-enriched miRNA patterns we observed, particularly in early-to-mid pregnancy, indicate that sedentary behavior may influence predicted target pathways related to RNA regulation, cell adhesion, and growth signaling; because these pathways are inferred from target prediction and database annotation rather than measured mRNA, protein, or phenotypic change, they should be interpreted as candidate downstream processes rather than confirmed developmental signals delivered through breast milk. Taken together, these results highlight sedentary behavior as a modifiable prenatal factor worth further investigation, raising hypothesis-generating clinical and translational questions that warrant examination in larger cohorts.

More broadly, EV-miRNAs represent a promising class of biomarkers for maternal behavior and physiology during gestation. Their stability, tissue specificity, and transportability make them attractive candidates for understanding how maternal lifestyle factors influence the molecular environment to which infants are exposed. Although therapeutic applications remain speculative, EV miRNAs are already being explored as targets for precision nutrition, drug delivery systems, and early-life programming interventions [53]. Collectively, these findings contribute to the growing field of nutrigenomics by identifying maternal movement behavior as a modifiable factor that shapes the bioactive molecular composition of a nutritional biofluid, with potential downstream consequences for infant health outcomes.

### 4.5. Strengths and Limitations

This study provides a prospective analysis relating maternal activity, measured repeatedly across pregnancy and the early postnatal period, to breast milk EV-enriched miRNA composition measured once, at 18 days postpartum. Strengths include device-based activity monitoring across all trimesters and the postnatal period, EV-enriched-fraction-specific small RNA sequencing, and rigorous data processing; repeated prenatal activity assessment enabled evaluation of whether associations with later milk miRNA composition differed across pregnancy stages, and the focus on extreme behavioral contrasts enhanced the ability to detect biologically meaningful signals.

Several factors temper how these findings should be read. The modest sample size (n = 11) limits generalizability and leaves DESeq2 dispersion estimates less stable than in larger studies; sensitivity analyses (Section 3.6) confirmed that re-estimating dispersion from the full analytic sample preserved the direction of effect for all quartile-identified miRNAs but with attenuated significance, indicating that the log_2_ fold-change magnitudes in Table 2 (in some cases 3–5) are best read as provisional estimates. The cohort also included uncontrolled confounders independently linked to milk composition, including gestational diabetes and a wide range of pre-pregnancy BMIs (mean: 26.8 ± 6.6 kg/m^2^, 30.8% classified as obese) and mixed parity; at this sample size we could not statistically adjust for these factors, so some of the associations attributed here to sedentary behavior may partly reflect this.

Additionally, because intravesicular miRNAs were not distinguished from surface-associated or co-isolated non-vesicular RNA (e.g., via an RNase protection assay with and without detergent), the sequenced miRNAs should be interpreted as originating from an EV-enriched milk fraction rather than as confirmed EV cargo. Similarly, TEM and imaging flow cytometry characterization of particle size and concentration were performed in a subset of donors (n = 4) as a proof-of-concept check on isolation quality, rather than across the full analytic sample; consequently, we cannot exclude the possibility that differences in EV concentration or recovery between sedentary-behavior groups contributed to the observed miRNA differences. Directly testing whether EV physical characteristics differ by maternal sedentary behavior is an important question for future studies but was outside the scope of this pilot investigation.

Despite these constraints, quartile-based grouping effectively highlighted meaningful behavioral contrasts and predicted target enrichment, providing a useful framework for identifying candidate pathways and establishing a foundation for future work. Larger cohorts, longitudinal designs with repeated milk sampling, longitudinal infant developmental follow-up, and experimental validation of miRNA–target interactions will be the essential next steps to clarify the biological relevance of these associations.

## 5. Conclusions

In this pilot study, maternal sedentary behavior during early and mid-pregnancy was associated with candidate, hypothesis-generating variation in human milk EV-enriched miRNA expression, particularly in predicted target pathways related to RNA regulation, cell adhesion, and growth signaling. As breast milk is the principal nutritional matrix consumed by infants during a critical window of development, this associative variation in its bioactive miRNA composition represents a plausible, preliminary nutrigenomic link between maternal prenatal lifestyle and early-life programming, though this remains to be established. These findings suggest that prolonged sedentary behavior may be associated with differences in HM EV-enriched miRNA expression and, potentially, the molecular signals delivered to the infant through breast milk. This study underscores the need for larger, mechanistic, and longitudinal studies linking maternal activity, HM EV miRNAs, and infant outcomes.

## Figures and Tables

**Table 1:** Sample cohort characteristics, n = 13.

Characteristics	n = 13
Health complications during pregnancy, n (%)	
Anemia	1 (7.7%)
Gestational diabetes	1 (7.7%)
Race/Ethnicity, n (%)	
White or Caucasian	11 (84.6%)
Multiracial	2 (15.4%)
Hispanic origin	2 (15.4%)
Minority status	4 (30.8%)
Pre-pregnancy BMI category, n (%)	
Normal	6 (46.2%)
Overweight	2 (15.4%)
Underweight	1 (7.7%)
Obese	4 (30.8%)
Pre-pregnancy BMI, mean ± SD (kg/m^2^)	26.8 ± 6.6
Parity, n (%)	
Nulliparous	8 (61.5%)
Multiparous	5 (38.5%)
Highest education level, n (%)	
Some College/Associate	1 (7.7%)
College Graduate	5 (38.5%)
Masters/Doctoral	7 (53.8%)
Annual household income, n (%)	
$25–49k	2 (15.4%)
$50–99k	5 (38.5%)
$100–149k	1 (7.7%)
$150–199k	1 (7.7%)
$200k+	4 (30.8%)
Maternal age, average mean ± SD (years)	29 ± 3.7

**Table 2:** List of differentially expressed (FDR ≤ 0.05) miRNAs in maternal minutes spent in prolonged sedentary behavior in trimesters one to three and postnatally (Q1 vs. Q3). miRNAs are ordered by adjusted *p*-value, with the smallest *p*-value at the top and the largest at the bottom. No miRNAs were differentially expressed postnatally. log_2_ fold-change values reflect the comparison with Q3 (highest prolonged sedentary behavior) as the reference group; positive values indicate higher expression in Q1 (lowest prolonged sedentary behavior).

**Trimester One: Number of Minutes in Prolonged Sedentary Behavior**			
**miRNA**	**baseMean**	**log_2_FoldChange**	**padj**
hsa-miR-7-5p	1267.5	−3.85	0.021
**Trimester Two: Number of Minutes in Prolonged Sedentary Behavior**			
**miRNA**	**baseMean**	**log_2_FoldChange**	**padj**
hsa-miR-511-5p	264.8	2.10	0.0012
hsa-miR-193a-3p	56.6	−3.68	0.0014
hsa-miR-12136	18.7	−5.87	0.0059
hsa-miR-148a-3p	164,168.5	1.78	0.0074
hsa-miR-33b-3p	56.0	−3.66	0.0074
hsa-miR-141-3p	913.7	−1.67	0.011
hsa-miR-375-3p	3191.5	1.49	0.011
hsa-miR-10a-5p	566.7	1.93	0.028
hsa-miR-429	627.1	−1.33	0.033
**Trimester Three: Number of Minutes in Prolonged Sedentary Behavior**			
**miRNA**	**baseMean**	**log_2_FoldChange**	**padj**
hsa-miR-148a-3p	83,244.9	1.26	0.027

## Data Availability

Data will be made publicly available in NCBI GEO (GSE335317) upon acceptance. All code scripts are available on GitHub (bpereira2022/Maternal-sedentary-behavior-and-breast-milk-EV-miRNA-across-pregnancy, v3.22.1).

## Supplementary Materials

The following supporting information can be downloaded at https://www.mdpi.com/article/10.3390/nu18193293/s1: Table S1: Sample sizes for differential gene expression analysis by maternal activity quartile groups; Table S2: Maternal activity patterns across pregnancy and postnatal; Table S3: Vesicle size distribution by sample and replicate for breastmilk EV preparations, alongside the Lipo100 synthetic vesicle standard; Table S4: Tetraspanin (TS mix-PE) and vFRed positivity by sample and replicate for breastmilk EV preparations, relative to the Lipo100 antigen-negative background control and EV reference preparation (positive control); Table S5: Pearson and Spearman correlations between PC1/PC2 scores of extracellular vesicle miRNA expression and continuous measures of MVPA and SED60+, by trimester and postnatal timepoint (n = 11); Table S6: PCA correlational analysis (Table S5) robustness to hsa-miR-148a-3p, with removal of hsa-miR-148a-3p prior to analysis; Table S7: List of significantly (FDR ≤ 0.05) differentially expressed miRNAs in maternal minutes spent in MVPA in trimesters one to three, and postpartum (Q3 vs. Q1); Table S8: Sensitivity analyses for all 17 miRNAs identified as differentially expressed (padj < 0.05) across quartile-based comparisons. log2FC and padj are from the primary quartile model; Table S9: DESeq2-normalized counts for each of the 17 miRNAs identified as differentially expressed (padj < 0.05), by participant, across all 8 comparisons; Table S10: Maximum Cook’s distance for each miRNA reported as differentially expressed (padj < 0.05) across all quartile-based comparisons, ranked highest to lowest; Table S11: PANTHER Gene Ontology (GO) biological process enrichment analysis of predicted miRNA target genes; Figure S1: Distribution of maternal movement behavior across pregnancy and postnatal by quartile; Figure S2: Comparison of sequencing metrics between lane one and lane two prior to merging; Figure S3: Plot of the top 20 contributors to PC1 and PC2; Figure S4: Individual normalized counts for the 9 miRNAs differentially expressed (padj < 0.05) between prolonged SED60+ behavior quartiles in Trimester 2; Figure S5: Maximum Cook’s distance per miRNA.
