# Supplementary material for: Maternal Sedentary Behavior and Breast Milk Extracellular Vesicle MicroRNA Across Pregnancy: A Pilot Study
Source: Nutrients. 2026 Oct 8;18(19):3293. doi: 10.3390/nu18193293 (PMC13667177; doi:10.3390/nu18193293)
Supplement: Supplementary file 1 [file nutrients-18-03293-s001.zip › nutrients-4443117-supplementary.pdf]

Supplementary Information

Figure S1. **Distribution of maternal movement behavior across pregnancy and postnatal by quartile.** Panels A-C show trimester-specific distributions of prolonged SED60+ behavior (left; average minutes per day spent in SED60+ bouts ≥60 minutes) and moderate-to-vigorous physical activity (MVPA; right; average minutes per day ≥3.0 METs accumulated in ≥30-second epochs) during trimester one (A), trimester two (B), and trimester three (C). Panel D shows postnatal distributions for prolonged SED60+ behavior (left) and active behavior (MVPA, right). Colors represent quartile groupings (Q1, interquartile range, Q3) defined separately within each period, as indicated in the legends. Boxplots illustrate the spread of observed values within each quartile and are provided for visualization of variable placement over time; no statistical comparisons are depicted. The number of samples within each quartile, for each maternal movement behavior and trimester, is next to quartile groupings.

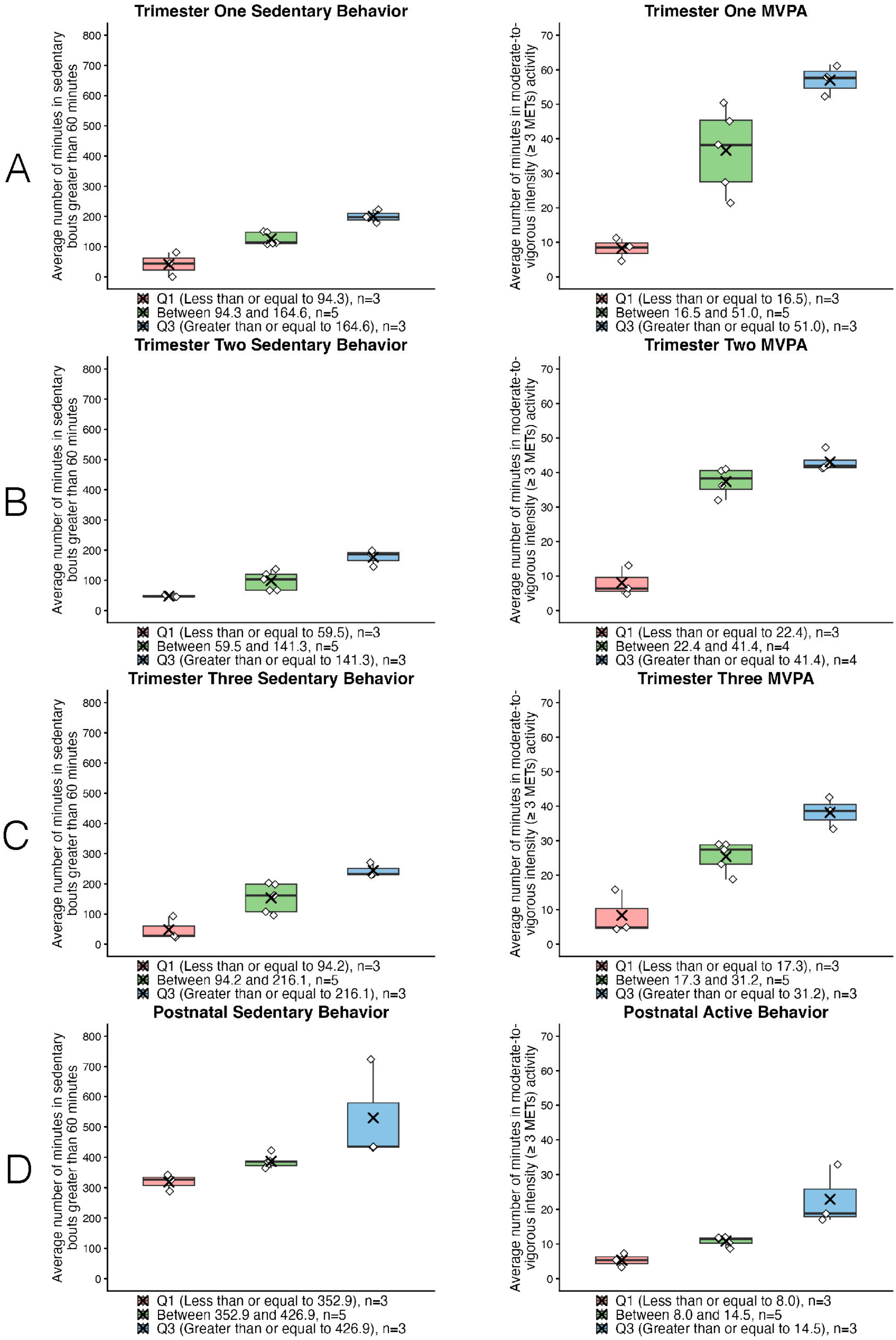
A
Trimester One Sedentary Behavior
Average number of minutes in sedentary bouts greater than 60 minutes
Q1 (Less than or equal to 94.3), n=3
Between 94.3 and 164.6, n=5
Q3 (Greater than or equal to 164.6), n=3
Trimester One MVPA
Average number of minutes in moderate-to-vigorous intensity (≥ 3 METs) activity
Q1 (Less than or equal to 16.5), n=3
Between 16.5 and 51.0, n=5
Q3 (Greater than or equal to 51.0), n=3
B
Trimester Two Sedentary Behavior
Average number of minutes in sedentary bouts greater than 60 minutes
Q1 (Less than or equal to 59.5), n=3
Between 59.5 and 141.3, n=5
Q3 (Greater than or equal to 141.3), n=3
Trimester Two MVPA
Average number of minutes in moderate-to-vigorous intensity (≥ 3 METs) activity
Q1 (Less than or equal to 22.4), n=3
Between 22.4 and 41.4, n=4
Q3 (Greater than or equal to 41.4), n=4
C
Trimester Three Sedentary Behavior
Average number of minutes in sedentary bouts greater than 60 minutes
Q1 (Less than or equal to 94.2), n=3
Between 94.2 and 216.1, n=5
Q3 (Greater than or equal to 216.1), n=3
Trimester Three MVPA
Average number of minutes in moderate-to-vigorous intensity (≥ 3 METs) activity
Q1 (Less than or equal to 17.3), n=3
Between 17.3 and 31.2, n=5
Q3 (Greater than or equal to 31.2), n=3
D
Postnatal Sedentary Behavior
Average number of minutes in sedentary bouts greater than 60 minutes
Q1 (Less than or equal to 352.9), n=3
Between 352.9 and 426.9, n=5
Q3 (Greater than or equal to 426.9), n=3
Postnatal Active Behavior
Average number of minutes in moderate-to-vigorous intensity (≥ 3 METs) activity
Q1 (Less than or equal to 8.0), n=3
Between 8.0 and 14.5, n=5
Q3 (Greater than or equal to 14.5), n=3

Figure S2. **Comparison of sequencing metrics between lane one and lane two prior to merging**. (a) Raw read counts per sample for both lanes. (b) Read counts after adapter and size trimming. (c) Final aligned mature miRNA counts for each lane. Bars represent counts for lane one (blue) and lane two (orange), showing high consistency across processing stages.

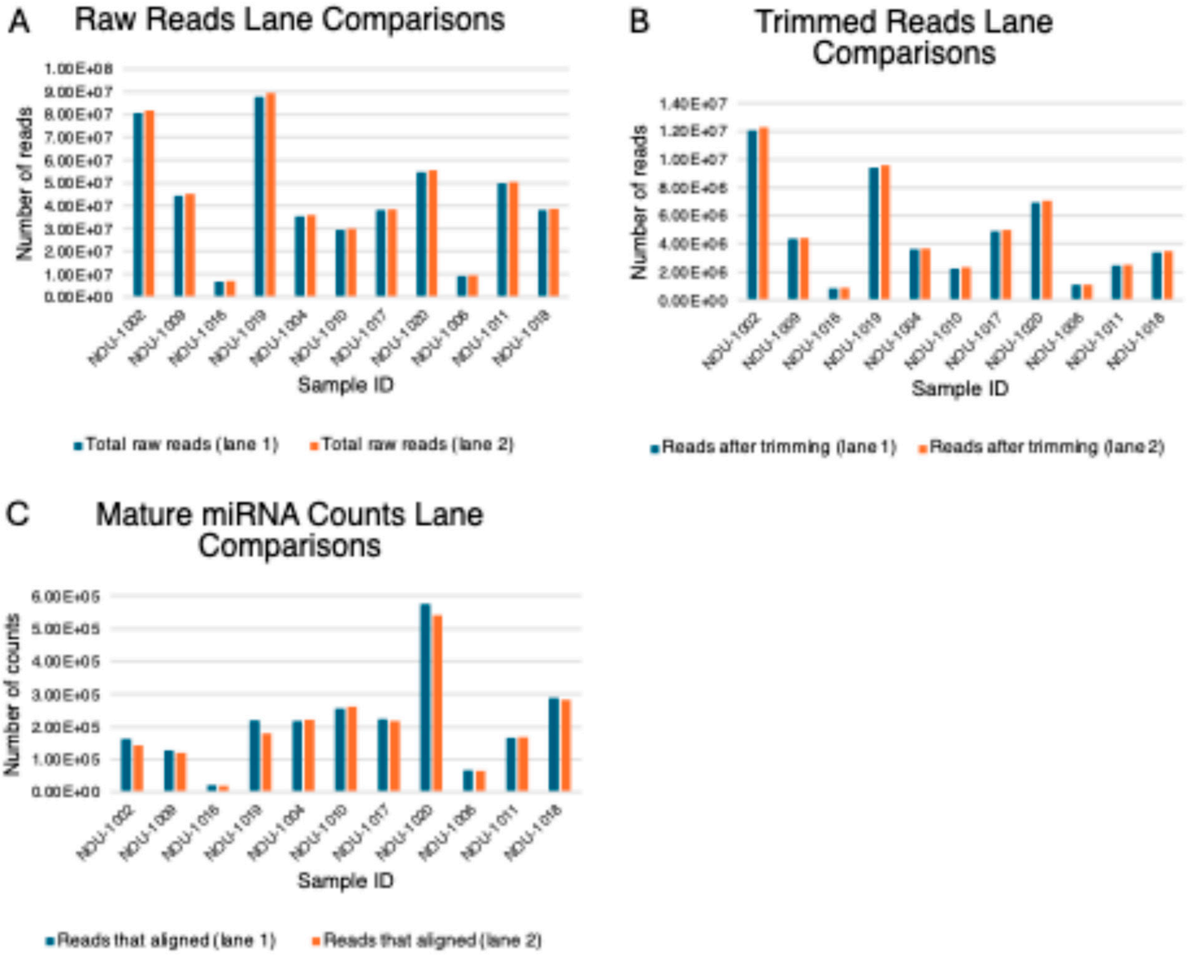

Figure S3. **Plot of the top 20 contributors to PC1 and PC2.** % contribution to each component was calculated as the PC loading squared times 100. hsa-miR-148a-3p, the miRNA that makes up 31.6% of the miRNA dataset, is highlighted in red.

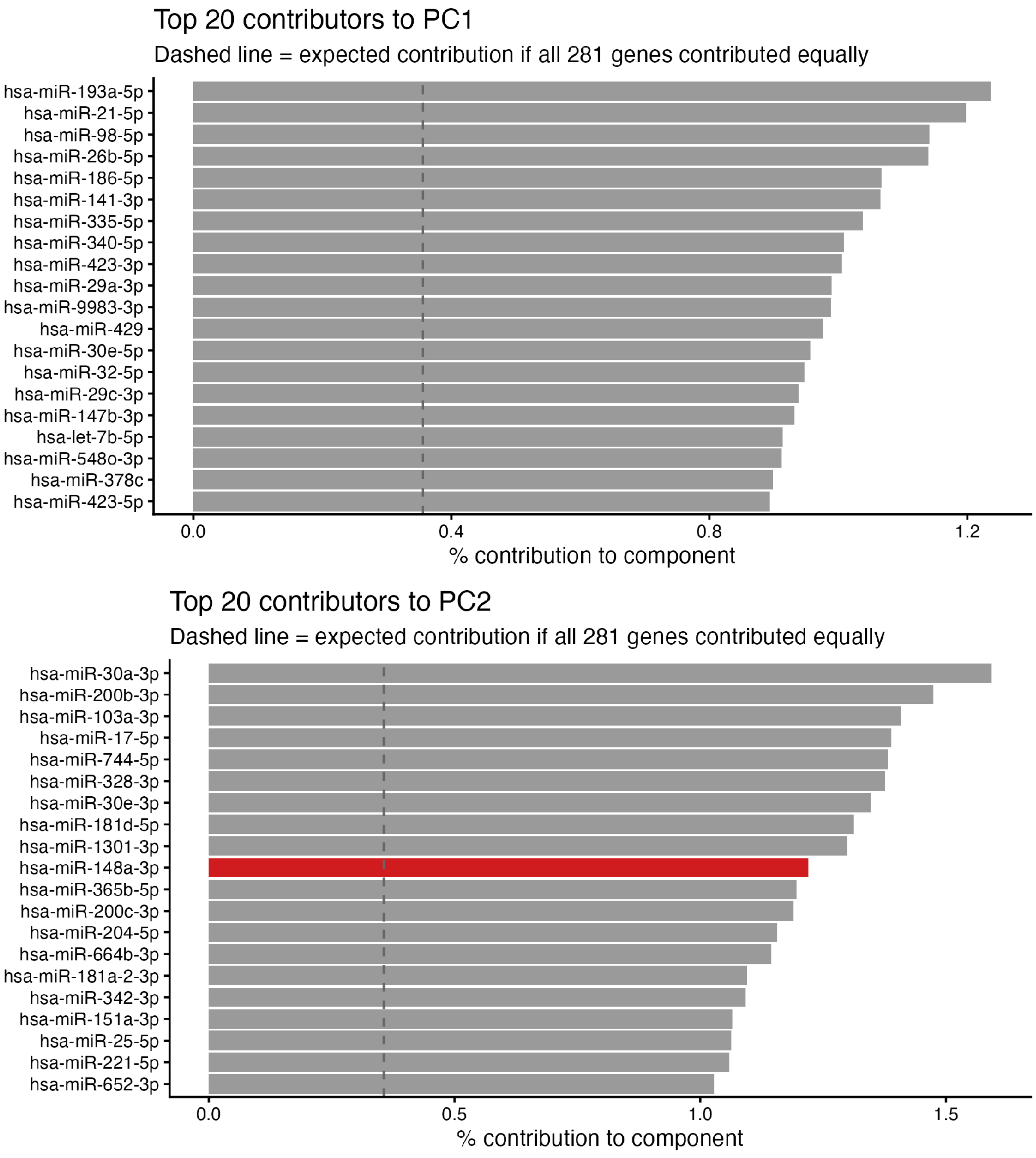

Figure S4. **Individual normalized counts for the 9 miRNAs differentially expressed (padj < 0.05) between prolonged SED60+ behavior quartiles in Trimester 2**. Counts are DESeq2-normalized; each point represents one participant, and horizontal black lines indicate the group mean. High (blue) = Q3, more SED60+; Low (red) = Q1, less SED60+. Note that y-axis scales differ across panels in absolute expression across miRNAs.

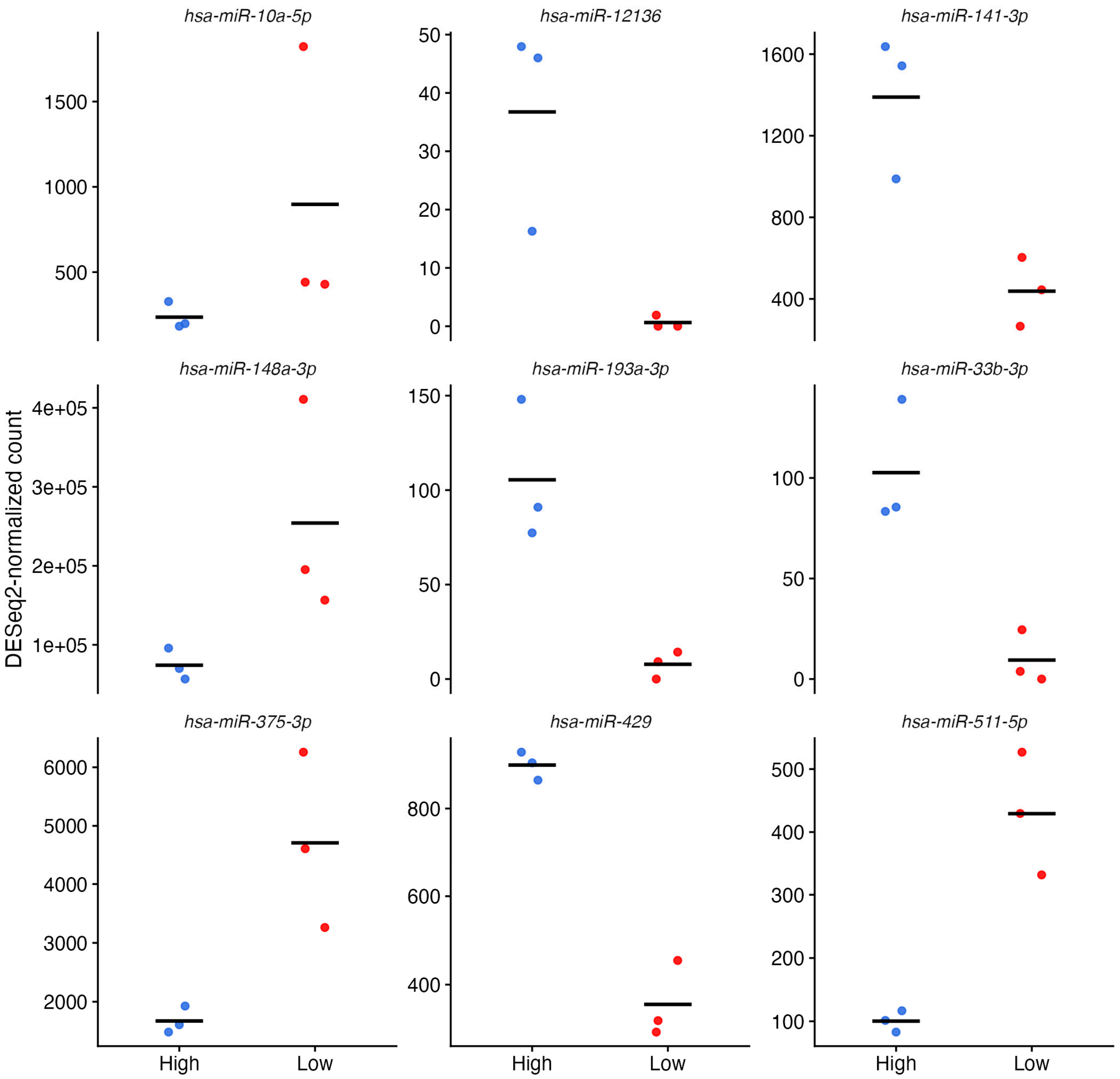

Figure S5. **Maximum Cook's distance per miRNA.** Each point is one miRNA tested in that comparison's primary quartile model; red points are miRNAs reported as differentially expressed (padj < 0.05). Dashed line = DESeq2's default automatic cutoff (F(2, n_samples − 2), 99th percentile), shown for reference only and not used as the flagging criterion (see Table S8).

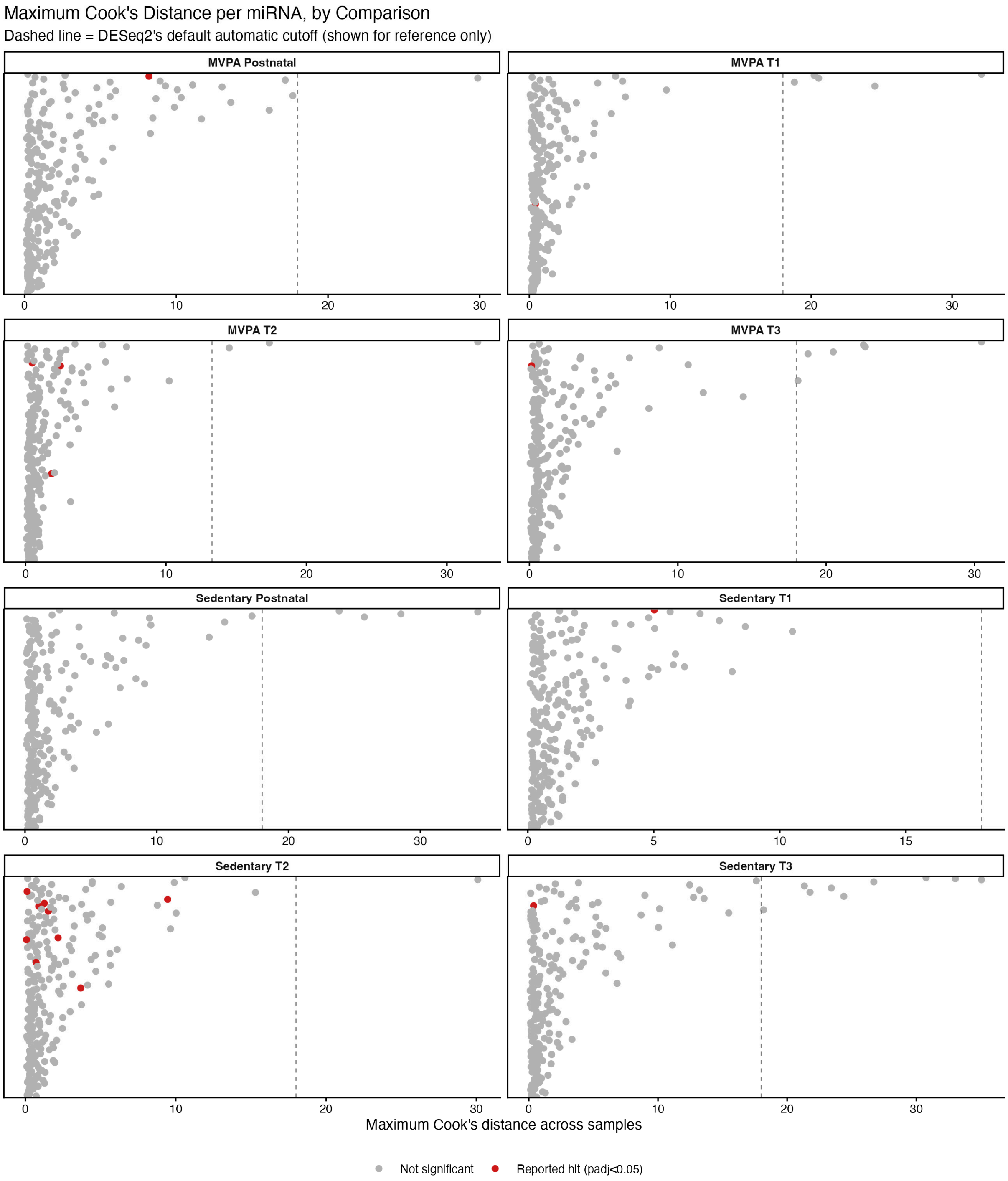

Table S1. **Sample sizes for differential gene expression analysis by maternal activity quartile groups.** Values indicate the total number of samples included in each comparison for lower quartile (Q1) and upper quartile (Q3) groups, calculated from the analytic sample (n = 11). Activity measures include moderate-to-vigorous physical activity (MVPA; minutes/day ≥3.0 METs) and prolonged SED60+ time (minutes/day in bouts ≥60 min; sed60num). Comparisons were performed separately for each time point (Trimester 1, Trimester 2, Trimester 3, and Postnatal).

**Comparison**	**Q3 total n**	**Q1 total n**
**MVPA Trimester 1**	3	3
**sed60num Trimester 1**	3	3
**MVPA Trimester 2**	4	3
**sed60num Trimester 2**	3	3
**MVPA Trimester 3**	3	3
**sed60num Trimester 3**	3	3
**MVPA Postnatal**	3	3
**sed60num Postnatal**	3	3

Table S2. **Maternal activity patterns across pregnancy and postnatal.** Data are shown for each time point (trimester one, two, three, and postnatal) as lower quartile (Q1), median, mean + standard deviation, and upper quartile (Q3). Units: minutes/day for SED60+ and MVPA. Quartiles were calculated from empirical distributions of the study cohort.

**SED60+: minutes/day in bouts ≥60 min**				
	**Trimester 1**	**Trimester 2**	**Trimester 3**	**Postnatal**
**Q1**	94.35	59.55	94.15	352.85
**Median**	113.6	103.4	161.2	385.9
**Mean (SD)**	123.18 (SD=66.10)	106.19 (SD=55.66)	149.37 (SD=84.75)	407.03 (SD=114.25)
**Q3**	164.55	141.3	216.05	426.85
**MVPA: minutes/day (≥3.0 METs)**				
	**Trimester 1**	**Trimester 2**	**Trimester 3**	**Postnatal**
**Q1**	16.5	22.45	17.3	8.04
**Median**	38.2	40.4	27.4	11.5
**Mean (SD)**	34.45 (SD=20.60)	31.45 (SD=15.61)	24.25 (SD=12.44)	12.65 (SD=8.10)
**Q3**	51	41.4	31.2	14.5

Table S3. **Vesicle size distribution by sample and replicate for breastmilk EV preparations, alongside the Lipo100 synthetic vesicle standard**. Median diameter, geometric mean, and coefficient of variation were calculated from calibrated vFRed fluorescence intensity on the ImageStream, gated on the Vesicles population.

Sample	Replicate	Median (nm)	Geo. Mean (nm)	CV (%)
NOU-1016	R1	96.27	105.69	52.75
NOU-1016	R2	96.04	100.37	38.56
NOU-1010	R1	96.25	105.12	46.52
NOU-1010	R2	96.26	105.33	47.36
NOU-1006	R1	96.19	102.31	46.78
NOU-1006	R2	96.28	103.66	47.64
NOU-1009	R1	97.74	110.28	47.49
NOU-1009	R2	103.31	115.16	51.57
Lipo100 (Control)	—	99.81	108.38	19.15

Table S4. **Tetraspanin (TS mix-PE) and vFRed positivity by sample and replicate for breastmilk EV preparations, relative to the Lipo100 antigen-negative background control and EV reference preparation (positive control)**. Event counts reflect the IDEAS-derived Vesicles gate population.

Sample	Replicate	Vesicles 2 (Events)	Vesicles 2 (% of total)	TS mix-PE+ (Events)	TS mix-PE+ (% of Vesicles 2)	vFRed+ (% of Vesicles 2)
NOU-1006	R1	11461	74.42	7845	68.45	87.12
NOU-1006	R2	14736	78.60	10402	70.59	90.85
NOU-1009	R1	25627	74.56	22228	86.74	92.07
NOU-1009	R2	32265	70.65	28227	87.48	93.00
NOU-1010	R1	14975	76.66	11826	78.97	88.04
NOU-1010	R2	16541	76.24	13018	78.70	87.56
NOU-1016	R1	19718	78.13	16589	84.13	90.09
NOU-1016	R2	21041	70.81	16932	80.47	89.09
EV Ref Prep	—	15612	99.37	13807	88.44	97.99
Lipo100	—	21661	80.18	5122	23.65	82.85

Table S5. **Pearson and Spearman correlations between PC1/PC2 scores of extracellular vesicle miRNA expression and continuous measures of MVPA and SED60+, by trimester and postnatal timepoint (n = 11).** Benjamini-Hochberg (BH) false discovery rate correction was applied separately within each 16-test family (Pearson, Spearman). Asterisks denote unadjusted p-values: *p < 0.05, **p < 0.01. T1 = trimester 1, T2 = trimester 2, T3 = trimester 2, PN = postnatal.

Activity	Timepoint	PC	Pearson r (p)	Spearman ρ (p)	BH-adj. Pearson p	BH-adj. Spearman p	n
**MVPA**	T1	PC1	-0.15 (p = 0.664)	-0.13 (p = 0.714)	0.914	0.917	11
**MVPA**	T1	PC2	-0.64 (p = 0.034)*	-0.65 (p = 0.037)*	0.182	0.148	11
**MVPA**	T2	PC1	0.18 (p = 0.596)	0.45 (p = 0.169)	0.914	0.450	11
**MVPA**	T2	PC2	-0.53 (p = 0.095)	-0.73 (p = 0.011)*	0.381	0.087	11
**MVPA**	T3	PC1	-0.05 (p = 0.888)	-0.04 (p = 0.924)	0.947	0.924	11
**MVPA**	T3	PC2	-0.35 (p = 0.288)	-0.35 (p = 0.286)	0.659	0.458	11
**MVPA**	PN	PC1	-0.42 (p = 0.194)	-0.41 (p = 0.214)	0.547	0.458	11
**MVPA**	PN	PC2	0.09 (p = 0.785)	0.06 (p = 0.860)	0.947	0.917	11
**SED60+**	T1	PC1	-0.25 (p = 0.459)	-0.36 (p = 0.273)	0.914	0.458	11
**SED60+**	T1	PC2	0.72 (p = 0.012)*	0.65 (p = 0.034)*	0.181	0.148	11
**SED60+**	T2	PC1	-0.14 (p = 0.676)	-0.36 (p = 0.273)	0.914	0.458	11
**SED60+**	T2	PC2	0.68 (p = 0.023)*	0.79 (p = 0.006)**	0.181	0.087	11
**SED60+**	T3	PC1	0.14 (p = 0.686)	0.11 (p = 0.755)	0.914	0.917	11
**SED60+**	T3	PC2	0.41 (p = 0.205)	0.45 (p = 0.163)	0.547	0.450	11
**SED60+**	PN	PC1	0.01 (p = 0.967)	0.11 (p = 0.755)	0.967	0.917	11
**SED60+**	PN	PC2	0.06 (p = 0.871)	-0.08 (p = 0.818)	0.947	0.917	11

Table S6. **PCA correlational analysis (Table S5) robustness to hsa-miR-148a-3p, with removal of hsa-miR-148a-3p prior to analysis.** Benjamini-Hochberg (BH) false discovery rate correction was applied separately within each 16-test family (Pearson, Spearman). T1 = trimester 1, T2 = trimester 2, T3 = trimester 2, PN = postnatal.

Activity	Timepoint	PC	Pearson_r	Pearson_p	Spearman	Spearman	Pearson BH	Spearman BH
**SED60+**	T1	PC1	-0.253	0.453	-0.364	0.273	0.906	0.458
**MVPA**	T1	PC1	-0.145	0.671	-0.127	0.714	0.917	0.917
**SED60+**	T2	PC1	-0.145	0.672	-0.364	0.273	0.917	0.458
**MVPA**	T2	PC1	0.182	0.592	0.446	0.169	0.917	0.450
**SED60+**	T3	PC1	0.137	0.688	0.109	0.755	0.917	0.917
**MVPA**	T3	PC1	-0.047	0.890	-0.036	0.924	0.950	0.924
**SED60+**	PP	PC1	0.014	0.967	0.109	0.755	0.967	0.917
**MVPA**	PP	PC1	-0.424	0.193	-0.409	0.214	0.535	0.458
**SED60+**	T1	PC2	0.722	0.012	0.655	0.034	0.179	0.148
**MVPA**	T1	PC2	-0.641	0.034	-0.645	0.037	0.179	0.148
**SED60+**	T2	PC2	0.670	0.024	0.791	0.006	0.179	0.087
**MVPA**	T2	PC2	-0.524	0.098	-0.729	0.011	0.393	0.087
**SED60+**	T3	PC2	0.418	0.201	0.455	0.163	0.535	0.450
**MVPA**	T3	PC2	-0.357	0.281	-0.355	0.286	0.643	0.458
**SED60+**	PN	PC2	0.058	0.865	-0.082	0.818	0.950	0.917
**MVPA**	PN	PC2	0.087	0.799	0.064	0.860	0.950	0.917

Table S7: **List of significantly (FDR ≤ 0.05) differentially expressed miRNAs in maternal minutes spent in MVPA in trimesters one to three, and postpartum (Q3 vs. Q1).** miRNAs are ordered by significance, with the most significantly differentially expressed at the top, and the least at the bottom. There were no significant differentially expressed miRNAs in postpartum. Log2 fold-change values reflect the comparison to Q1 (lowest MVPA) as the reference group; positive values indicate higher expression in Q3 (highest MVPA/most active behavior). Sample sizes are provided in Table S1.

**Trimester one: MVPA**			
miRNA	baseMean	log2FoldChange	padj
hsa-miR-143-3p	467.2	2.70	0.016
**Trimester two: MVPA**			
miRNA	baseMean	log2FoldChange	padj
hsa-miR-143-3p	400.13	2.69	0.00062
hsa-miR-10a-5p	501.41	2.23	0.0070
hsa-miR-151a-3p	9383.63	1.81	0.046
hsa-miR-148a-3p	136123.45	1.67	0.050
**Trimester three: MVPA**			
miRNA	baseMean	log2FoldChange	padj
hsa-miR-516b-5p	32.2	2.69	0.047
**Postpartum: MVPA**			
miRNA	baseMean	log2FoldChange	padj
hsa-miR-223-3p	468.6	5.43	0.0059

Table S8. **Sensitivity analyses for all 17 miRNAs identified as differentially expressed (padj < 0.05) across quartile-based comparisons. log2FC and padj are from the primary quartile model.** Continuous_direction (cont. dir.) indicates whether a continuous-exposure model (full n = 11, direction of effect only) agreed in direction with the quartile model. padj_candidateFDR is the Benjamini-Hochberg-adjusted p-value from the continuous model, restricted to this pre-specified candidate list. padj_fulldispersion is significance when DESeq2 dispersion/size-factor estimation used the full n = 11 rather than the extreme quartiles alone (n = 6-7), retaining the same contrast. Exclusion_Status indicates whether the finding was robust to removal of hsa-miR-148a-3p from the count matrix ("N/A" for hsa-miR-148a-3p itself, which cannot be tested against its own exclusion).

miRNA	Comparison	log2FC	padj	cont. dir.	padj_candidateFDR	padj_fulldispersion	Exclusion_Status
hsa-miR-7-5p	SED60+ T1	-3.85	0.022	Yes	<0.001	0.091	Robust
hsa-miR-511-5p	SED60+ T2	2.1	0.001	Yes	0.050	0.054	Robust
hsa-miR-193a-3p	SED60+ T2	-3.68	0.001	Yes	0.110	0.033	Robust
hsa-miR-12136	SED60+ T2	-5.87	0.006	Yes	0.111	0.004	Robust
hsa-miR-148a-3p	SED60+ T2	1.78	0.007	Yes	0.023	0.099	N/A
hsa-miR-33b-3p	SED60+ T2	-3.66	0.007	Yes	0.048	0.056	Robust
hsa-miR-141-3p	SED60+ T2	-1.67	0.011	Yes	0.882	0.506	Robust
hsa-miR-375-3p	SED60+ T2	1.49	0.011	Yes	0.110	0.099	Robust
hsa-miR-10a-5p	SED60+ T2	1.93	0.028	Yes	0.023	0.214	Robust
hsa-miR-429	SED60+ T2	-1.33	0.033	Yes	0.271	0.316	Robust
hsa-miR-148a-3p	SED60+ T3	1.26	0.027	Yes	0.661	0.702	N/A
hsa-miR-143-3p	MVPA T1	2.7	0.016	Yes	0.001	0.603	Robust
hsa-miR-143-3p	MVPA T2	2.69	0.001	Yes	0.017	0.174	Robust
hsa-miR-10a-5p	MVPA T2	2.23	0.007	Yes	0.006	0.174	Robust
hsa-miR-151a-3p	MVPA T2	1.81	0.046	Yes	0.017	0.174	Robust
hsa-miR-516b-5p	MVPA T3	2.69	0.047	Yes	0.033	0.206	Lost
hsa-miR-223-3p	MVPA Postnatal	5.43	0.006	Yes	<0.001	0.912	Robust

Table S9. **DESeq2-normalized counts for each of the 17 miRNAs identified as differentially expressed (padj < 0.05), by participant, across all 8 comparisons.** Group reflects quartile membership (High = Q3, Low = Q1) for the corresponding comparison.

miRNA	Sample ID	NormalizedCount	Group	Timepoint
hsa-miR-7-5p	NOU-1009	6489.2	High	SED60+ Trimester 1
hsa-miR-7-5p	NOU-1010	100.7	High	SED60+ Trimester 1
hsa-miR-7-5p	NOU-1020	522.5	High	SED60+ Trimester 1
hsa-miR-7-5p	NOU-1002	189.2	Low	SED60+ Trimester 1
hsa-miR-7-5p	NOU-1006	66.1	Low	SED60+ Trimester 1
hsa-miR-7-5p	NOU-1017	237.2	Low	SED60+ Trimester 1
hsa-miR-10a-5p	NOU-1009	197.9	High	SED60+ Trimester 2
hsa-miR-10a-5p	NOU-1018	182.7	High	SED60+ Trimester 2
hsa-miR-10a-5p	NOU-1020	327.6	High	SED60+ Trimester 2
hsa-miR-10a-5p	NOU-1006	441.1	Low	SED60+ Trimester 2
hsa-miR-10a-5p	NOU-1017	428.7	Low	SED60+ Trimester 2
hsa-miR-10a-5p	NOU-1019	1822.5	Low	SED60+ Trimester 2
hsa-miR-12136	NOU-1009	46.0	High	SED60+ Trimester 2
hsa-miR-12136	NOU-1018	16.3	High	SED60+ Trimester 2
hsa-miR-12136	NOU-1020	47.9	High	SED60+ Trimester 2
hsa-miR-12136	NOU-1006	0.0	Low	SED60+ Trimester 2
hsa-miR-12136	NOU-1017	0.0	Low	SED60+ Trimester 2
hsa-miR-12136	NOU-1019	1.9	Low	SED60+ Trimester 2
hsa-miR-141-3p	NOU-1009	1542.8	High	SED60+ Trimester 2
hsa-miR-141-3p	NOU-1018	988.4	High	SED60+ Trimester 2
hsa-miR-141-3p	NOU-1020	1637.0	High	SED60+ Trimester 2
hsa-miR-141-3p	NOU-1006	603.4	Low	SED60+ Trimester 2
hsa-miR-141-3p	NOU-1017	444.6	Low	SED60+ Trimester 2
hsa-miR-141-3p	NOU-1019	266.1	Low	SED60+ Trimester 2
hsa-miR-148a-3p	NOU-1009	56679.3	High	SED60+ Trimester 2
hsa-miR-148a-3p	NOU-1018	70024.5	High	SED60+ Trimester 2
hsa-miR-148a-3p	NOU-1020	95816.8	High	SED60+ Trimester 2
hsa-miR-148a-3p	NOU-1006	195199.0	Low	SED60+ Trimester 2
hsa-miR-148a-3p	NOU-1017	156572.0	Low	SED60+ Trimester 2
hsa-miR-148a-3p	NOU-1019	410719.2	Low	SED60+ Trimester 2
hsa-miR-193a-3p	NOU-1009	90.9	High	SED60+ Trimester 2
hsa-miR-193a-3p	NOU-1018	77.4	High	SED60+ Trimester 2
hsa-miR-193a-3p	NOU-1020	148.0	High	SED60+ Trimester 2
hsa-miR-193a-3p	NOU-1006	9.2	Low	SED60+ Trimester 2
hsa-miR-193a-3p	NOU-1017	14.3	Low	SED60+ Trimester 2
hsa-miR-193a-3p	NOU-1019	0.0	Low	SED60+ Trimester 2
hsa-miR-33b-3p	NOU-1009	139.1	High	SED60+ Trimester 2
hsa-miR-33b-3p	NOU-1018	85.5	High	SED60+ Trimester 2
hsa-miR-33b-3p	NOU-1020	83.3	High	SED60+ Trimester 2
hsa-miR-33b-3p	NOU-1006	24.5	Low	SED60+ Trimester 2
hsa-miR-33b-3p	NOU-1017	0.0	Low	SED60+ Trimester 2
hsa-miR-33b-3p	NOU-1019	3.8	Low	SED60+ Trimester 2
hsa-miR-375-3p	NOU-1009	1926.9	High	SED60+ Trimester 2
hsa-miR-375-3p	NOU-1018	1608.8	High	SED60+ Trimester 2
hsa-miR-375-3p	NOU-1020	1484.2	High	SED60+ Trimester 2
hsa-miR-375-3p	NOU-1006	4609.7	Low	SED60+ Trimester 2
hsa-miR-375-3p	NOU-1017	3261.7	Low	SED60+ Trimester 2
hsa-miR-375-3p	NOU-1019	6257.9	Low	SED60+ Trimester 2

hsa-miR-429	NOU-1009	864.5	High	SED60+ Trimester 2
hsa-miR-429	NOU-1018	903.9	High	SED60+ Trimester 2
hsa-miR-429	NOU-1020	928.1	High	SED60+ Trimester 2
hsa-miR-429	NOU-1006	318.5	Low	SED60+ Trimester 2
hsa-miR-429	NOU-1017	454.7	Low	SED60+ Trimester 2
hsa-miR-429	NOU-1019	292.7	Low	SED60+ Trimester 2
hsa-miR-511-5p	NOU-1009	116.6	High	SED60+ Trimester 2
hsa-miR-511-5p	NOU-1018	83.0	High	SED60+ Trimester 2
hsa-miR-511-5p	NOU-1020	101.4	High	SED60+ Trimester 2
hsa-miR-511-5p	NOU-1006	526.8	Low	SED60+ Trimester 2
hsa-miR-511-5p	NOU-1017	331.4	Low	SED60+ Trimester 2
hsa-miR-511-5p	NOU-1019	429.5	Low	SED60+ Trimester 2
hsa-miR-148a-3p	NOU-1009	39228.3	High	SED60+ Trimester 3
hsa-miR-148a-3p	NOU-1016	58351.6	High	SED60+ Trimester 3
hsa-miR-148a-3p	NOU-1018	49420.6	High	SED60+ Trimester 3
hsa-miR-148a-3p	NOU-1004	104476.5	Low	SED60+ Trimester 3
hsa-miR-148a-3p	NOU-1006	137445.2	Low	SED60+ Trimester 3
hsa-miR-148a-3p	NOU-1017	110547.1	Low	SED60+ Trimester 3
hsa-miR-143-3p	NOU-1002	1423.9	High	MVPA Trimester 1
hsa-miR-143-3p	NOU-1006	624.6	High	MVPA Trimester 1
hsa-miR-143-3p	NOU-1020	380.0	High	MVPA Trimester 1
hsa-miR-143-3p	NOU-1009	225.4	Low	MVPA Trimester 1
hsa-miR-143-3p	NOU-1010	62.0	Low	MVPA Trimester 1
hsa-miR-143-3p	NOU-1018	87.2	Low	MVPA Trimester 1
hsa-miR-10a-5p	NOU-1002	632.9	High	MVPA Trimester 2
hsa-miR-10a-5p	NOU-1006	396.7	High	MVPA Trimester 2
hsa-miR-10a-5p	NOU-1017	384.3	High	MVPA Trimester 2
hsa-miR-10a-5p	NOU-1019	1613.3	High	MVPA Trimester 2
hsa-miR-10a-5p	NOU-1009	172.7	Low	MVPA Trimester 2
hsa-miR-10a-5p	NOU-1010	138.5	Low	MVPA Trimester 2
hsa-miR-10a-5p	NOU-1018	171.5	Low	MVPA Trimester 2
hsa-miR-143-3p	NOU-1002	1098.4	High	MVPA Trimester 2
hsa-miR-143-3p	NOU-1006	498.6	High	MVPA Trimester 2
hsa-miR-143-3p	NOU-1017	370.7	High	MVPA Trimester 2
hsa-miR-143-3p	NOU-1019	540.0	High	MVPA Trimester 2
hsa-miR-143-3p	NOU-1009	178.3	Low	MVPA Trimester 2
hsa-miR-143-3p	NOU-1010	47.0	Low	MVPA Trimester 2
hsa-miR-143-3p	NOU-1018	67.8	Low	MVPA Trimester 2
hsa-miR-151a-3p	NOU-1002	11259.7	High	MVPA Trimester 2
hsa-miR-151a-3p	NOU-1006	11573.4	High	MVPA Trimester 2
hsa-miR-151a-3p	NOU-1017	5199.2	High	MVPA Trimester 2
hsa-miR-151a-3p	NOU-1019	26049.5	High	MVPA Trimester 2
hsa-miR-151a-3p	NOU-1009	4788.7	Low	MVPA Trimester 2
hsa-miR-151a-3p	NOU-1010	3670.2	Low	MVPA Trimester 2
hsa-miR-151a-3p	NOU-1018	3144.8	Low	MVPA Trimester 2
hsa-miR-516b-5p	NOU-1006	39.9	High	MVPA Trimester 3
hsa-miR-516b-5p	NOU-1016	58.4	High	MVPA Trimester 3
hsa-miR-516b-5p	NOU-1020	68.7	High	MVPA Trimester 3
hsa-miR-516b-5p	NOU-1009	6.9	Low	MVPA Trimester 3
hsa-miR-516b-5p	NOU-1017	11.2	Low	MVPA Trimester 3
hsa-miR-516b-5p	NOU-1018	7.8	Low	MVPA Trimester 3
hsa-miR-223-3p	NOU-1004	2601.5	High	MVPA Postnatal
hsa-miR-223-3p	NOU-1010	136.8	High	MVPA Postnatal

hsa-miR-223-3p	NOU-1020	6.9	High	MVPA Postnatal
hsa-miR-223-3p	NOU-1016	34.6	Low	MVPA Postnatal
hsa-miR-223-3p	NOU-1018	7.0	Low	MVPA Postnatal
hsa-miR-223-3p	NOU-1019	24.9	Low	MVPA Postnatal

Table S10. **Maximum Cook's distance for each miRNA reported as differentially expressed (padj < 0.05) across all quartile-based comparisons, ranked highest to lowest.** default_cutoff reflects DESeq2's automatic threshold (99th percentile of F(2, n_samples − 2)) and is shown for reference only; it was not used as the flagging criterion. p95_comparison is the 95th percentile of maxCooks across all genes tested within that comparison, used as a numerical anchor for visual inspection of Figure S5; flagged = maxCooks ≥ p95_comparison.

miRNA	maxCooks	Comparison	default_cutoff	p95_comparison	n_samples	flagged
hsa-miR-10a-5p	9.46	Sedentary T2	18.0	5.65	6	Yes
hsa-miR-223-3p	8.2	MVPA Postnatal	18.0	9.18	6	No
hsa-miR-7-5p	5.02	Sedentary T1	18.0	4.99	6	Yes
hsa-miR-33b-3p	3.68	Sedentary T2	18.0	5.65	6	No
hsa-miR-10a-5p	2.48	MVPA T2	13.27	4.26	7	No
hsa-miR-12136	2.17	Sedentary T2	18.0	5.65	6	No
hsa-miR-143-3p	1.85	MVPA T2	13.27	4.26	7	No
hsa-miR-193a-3p	1.52	Sedentary T2	18.0	5.65	6	No
hsa-miR-148a-3p	1.26	Sedentary T2	18.0	5.65	6	No
hsa-miR-141-3p	0.89	Sedentary T2	18.0	5.65	6	No
hsa-miR-429	0.71	Sedentary T2	18.0	5.65	6	No
hsa-miR-151a-3p	0.47	MVPA T2	13.27	4.26	7	No
hsa-miR-143-3p	0.43	MVPA T1	18.0	4.62	6	No
hsa-miR-148a-3p	0.37	Sedentary T3	18.0	14.33	6	No
hsa-miR-516b-5p	0.14	MVPA T3	18.0	6.69	6	No
hsa-miR-375-3p	0.11	Sedentary T2	18.0	5.65	6	No
hsa-miR-511-5p	0.07	Sedentary T2	18.0	5.65	6	No

Table S11: **PANTHER Gene Ontology (GO) biological process enrichment analysis of predicted miRNA target genes.** The top 20 significantly enriched GO biological processes among predicted targets of trimester-two sedentary-behavior-associated miRNAs, identified using the PANTHER Classification System and ranked by fold enrichment, grouped into five umbrella categories. For each GO term, the table lists the umbrella category, the number of genes annotated to the term in the reference list, the number of observed genes in the query (predicted target) set, the expected gene count, the fold enrichment, and the Bonferroni-corrected p-value (≤ 0.05). All listed processes reflect positive (overrepresented) enrichment per PANTHER's overrepresentation test.

GO Biological Process Complete Term	Umbrella Category	Ref_Count	Query_Count	Expected	Fold Enrichment	Bonferroni
**regulation of mRNA metabolic process**	RNA Regulation & Post-transcriptional Control	317	65	27.58	2.36	4.06E-07
**regulation of TOR signaling**	Growth & Nutrient Signaling	150	31	13.05	2.38	0.0438
**regulation of cell cycle G1/S phase transition**	Cell Cycle Regulation	152	32	13.22	2.42	0.0235
**regulation of mRNA catabolic process**	RNA Regulation & Post-transcriptional Control	207	47	18.01	2.61	5.37E-06
**regulation of G1/S transition of mitotic cell cycle**	Cell Cycle Regulation	131	30	11.4	2.63	0.00526
**regulation of RNA stability**	RNA Regulation & Post-transcriptional Control	191	44	16.62	2.65	1.16E-05
**regulation of cell-matrix adhesion**	Cell Adhesion & Cytoskeletal Organization	112	26	9.74	2.67	0.0219
**regulation of mRNA stability**	RNA Regulation & Post-transcriptional Control	181	42	15.75	2.67	2.16E-05
**regulation of TORC1 signaling**	Growth & Nutrient Signaling	102	25	8.87	2.82	0.0117
**positive regulation of mRNA metabolic process**	RNA Regulation & Post-transcriptional Control	122	30	10.61	2.83	0.001
**positive regulation of mRNA catabolic process**	RNA Regulation & Post-transcriptional Control	105	27	9.14	2.96	0.00159
**regulation of cell-substrate junction organization**	Cell Adhesion & Cytoskeletal Organization	68	21	5.92	3.55	0.00127
**negative regulation of TORC1 signaling**	Growth & Nutrient Signaling	51	16	4.44	3.61	0.0297
**regulation of focal adhesion assembly**	Cell Adhesion & Cytoskeletal Organization	62	20	5.39	3.71	0.00111
**regulation of cell-substrate junction assembly**	Cell Adhesion & Cytoskeletal Organization	62	20	5.39	3.71	0.00111
**negative regulation of translation**	RNA Regulation & Post-transcriptional Control	122	41	10.61	3.86	7.17E-11
**peptidyl-serine phosphorylation**	Protein Phosphorylation & Signaling	36	13	3.13	4.15	0.0419

**regulation of hippo signaling**	Growth & Nutrient Signaling	47	17	4.09	4.16	0.00147
**regulation of nuclear-transcribed mRNA catabolic process, deadenylation-dependent decay**	RNA Regulation & Post-transcriptional Control	24	13	2.09	6.23	0.000122
**miRNA-mediated gene silencing by inhibition of translation**	RNA Regulation & Post-transcriptional Control	18	10	1.57	6.39	0.00433
